# The Impact of Mobile Technology-Delivered Interventions on Youth Well-being: Systematic Review and 3-Level Meta-analysis

**DOI:** 10.2196/34254

**Published:** 2022-07-29

**Authors:** Colleen S Conley, Elizabeth B Raposa, Kate Bartolotta, Sarah E Broner, Maya Hareli, Nicola Forbes, Kirsten M Christensen, Mark Assink

**Affiliations:** 1 Department of Psychology Loyola University Chicago Chicago, IL United States; 2 Department of Psychology Fordham University Bronx, NY United States; 3 Department of Psychology University of Massachusetts Boston Boston, MA United States; 4 Faculty of Social and Behavioural Sciences University of Amsterdam Amsterdam Netherlands

**Keywords:** meta-analysis, mental health, well-being, intervention, treatment, youth, technology, smartphone, mobile phone, app, mobile health

## Abstract

**Background:**

Rates of mental health problems among youth are high and rising, whereas treatment seeking in this population remains low. Technology-delivered interventions (TDIs) appear to be promising avenues for broadening the reach of evidence-based interventions for youth well-being. However, to date, meta-analytic reviews on youth samples have primarily been limited to computer and internet interventions, whereas meta-analytic evidence on mobile TDIs (mTDIs), largely comprising mobile apps for smartphones and tablets, have primarily focused on adult samples.

**Objective:**

This study aimed to evaluate the effectiveness of mTDIs for a broad range of well-being outcomes in unselected, at-risk, and clinical samples of youth.

**Methods:**

The systematic review used 5 major search strategies to identify 80 studies evaluating 83 wellness- and mental health-focused mTDIs for 19,748 youth (mean age 2.93-26.25 years). We conducted a 3-level meta-analysis on the full sample and a subsample of the 38 highest-quality studies.

**Results:**

Analyses demonstrated significant benefits of mTDIs for youth both at posttest (*g*=0.27) and follow-up (range 1.21-43.14 weeks; *g*=0.26) for a variety of psychosocial outcomes, including general well-being and distress, symptoms of diverse psychological disorders, psychosocial strategies and skills, and health-related symptoms and behaviors. Effects were significantly moderated by the type of comparison group (strongest for no intervention, followed by inert placebo or information-only, and only marginal for clinical comparison) but only among the higher-quality studies. With respect to youth characteristics, neither gender nor pre-existing mental health risk level (not selected for risk, at-risk, or clinical) moderated effect sizes; however, effects increased with the age of youth in the higher-quality studies. In terms of intervention features, mTDIs in these research studies were effective regardless of whether they included various technological features (eg, tailoring, social elements, or gamification) or support features (eg, orientation, reminders, or coaching), although the use of mTDIs in a research context likely differs in important ways from their use when taken up through self-motivation, parent direction, peer suggestion, or clinician referral. Only mTDIs with a clear prescription for frequent use (ie, at least once per week) showed significant effects, although this effect was evident only in the higher-quality subsample. Moderation analyses did not detect statistically significant differences in effect sizes based on the prescribed duration of mTDI use (weeks or sessions), and reporting issues in primary studies limited the analysis of completed duration, thereby calling for improved methodology, assessment, and reporting to clarify true effects.

**Conclusions:**

Overall, this study’s findings demonstrate that youth can experience broad and durable benefits of mTDIs, delivered in a variety of ways, and suggest directions for future research and development of mTDIs for youth, particularly in more naturalistic and ecologically valid settings.

## Introduction

### Youth Mental Health Needs

Rates of mental health problems among youth, including children, adolescents, and young adults, are alarmingly high and appear to have risen in recent decades [1,2]. Rates of impulse control disorders (eg, attention deficit hyperactivity disorder and conduct disorder) and some anxiety disorders begin rising as early as the age of 4 years, with sharp increases in the prevalence of anxiety, mood, and substance use disorders across adolescence and young adulthood [3]. Indeed, nationally representative samples of adolescents and young adults show that >40% of youth in this age range experience psychological disorders in a given year and lifetime prevalence rates are estimated to approach 60% [4,5]. Beyond diagnosable mental disorders, many youths struggle with a diverse array of subclinical emotional, behavioral, interpersonal, and academic challenges [6-10].

Despite the high prevalence of youth facing mental health problems, only one-third to one-half of those in need receive mental health treatment [11-14], and treatment rates are even lower among low-income youth [15] and those with marginalized racial and ethnic identities [13,16,17]. This treatment gap is also evident among college students [11], which is notable given that these youth often have convenient access on campus to no- or low-cost mental health services [18,19].

Youth and families face several barriers to receiving mental health services [20-24]. Many lack knowledge and awareness of common mental health problems and may assume that certain behavioral or emotional issues are simply temporary phases or difficulties they can address on their own. These types of assumptions may be compounded by the stigma about mental illness and psychological services within the youth’s family, culture, or broader community [25,26]. Many youths and caregivers also lack knowledge of available evidence-based treatments for mental health problems. Furthermore, several structural barriers severely limit the access of many youths and families to culturally sensitive, effective mental health care. Low-cost, evidence-based treatments for youth mental health problems are not available in many underserved communities across the world, including low-income rural and urban areas and countries with limited health care infrastructure. Even when such treatments are available, families may lack the time or resources needed to travel and take advantage of these treatments [27-30].

### Technology’s Role in Youth Mental Health

Although efforts should continue to address the barriers that prevent formal services by qualified mental health professionals, it is also important to consider alternative ways of fulfilling the unmet mental health needs among youth. Mobile technology–delivered interventions (mTDIs), including mental health content delivered via mobile phones, tablets, and wearable smart devices (eg, watches, glasses, and virtual reality [VR] headsets), are potential ways of meeting this need. As of 2018, youth smartphone ownership and use were remarkably high, with 95% of teenagers having access to smartphones [31]. In 2016, the average age of first owning a smartphone was 10 years in the United States [32], and younger children commonly have access to smart devices through parents, siblings, or schools that provide tablets and other mobile devices to students. These mobile devices may be overlooked conduits for mental health information. Indeed, a recent survey of teenagers and young adults [33] reported that among those with moderate to severe depressive symptoms, 90% had searched the internet for information about mental health, and 38% used a mental health app. Parents also often use the internet for resources on health-related issues among their young children [34].

Technology can offer easy ways of connecting with mental health resources, such as mood-enhancing and skill-building apps purported to improve mental health. The ubiquitous, self-guided nature of such technology-delivered tools makes them appealing alternatives for those who are limited by access to, or trust in, formal mental health services [35]. Key themes in a recent review of research on internet-based help seeking for mental health difficulties among young people (aged up to 25 years) [36] showed that youth frequently engaged in technology-based (eg, internet-based) help seeking late at night (when traditional in-person mental health services are typically not available) and that youth endorsed several specific benefits of seeking help this way, including anonymity and privacy; lower perceived stigma and judgment; accessibility, including in times of crisis; and connection to others with similar experiences, which can foster a sense of community and acceptance.

### Technology-Delivered Interventions for Youth

#### Potential and Pitfalls

Although mTDIs have great potential to improve access to evidence-based mental health content, it is important to carefully evaluate their effects when it comes to mental health care, especially for youth. In contrast to computer- and internet-based technology-delivered interventions (TDIs), which are typically developed by clinicians and researchers to incorporate comprehensive and evidence-based treatment methods that parallel professional psychotherapy, *mobile* TDIs often lack such comprehensive, evidence-based principles while also introducing privacy and safety concerns [37]. The rapidly developing and competitive mobile app marketplace also poses some challenges in connecting evidence-based mental health practices with marketable and engaging mobile technology. Commercially available apps are typically designed by technology companies outside of the health care industry [38,39] with the aim of being engaging and attractive, thereby prioritizing appealing features such as design and gamification over evidence-based clinical techniques [40,41]. In contrast, research-developed apps prioritize evidence-based content and rigorous trials, which slows widespread availability as it can take 2 decades from conceptualization to public dissemination [37,42], leaving a gap between research-tested and commercially available mTDIs. Indeed, recent reviews of the content of >10,000 purported mental health apps in the commercial marketplace have noted that a vast majority have not gone through rigorous intervention development and testing [43] and are lacking or inconsistent with evidence-based psychotherapy principles [44], including apps specifically targeting youth mental health [40,45]. Although research trials have demonstrated promising findings for some mTDIs, the heterogeneity and poor quality of certain mTDIs and studies have led to inconclusive evidence across outcomes [46], which makes the role of these interventions in mental health services less clear [47]. Finally, mental health technologies generally have low rates of engagement and adherence [35,43,48-50]. Thus, a key question for this emerging area of research is how mTDIs can best be designed, prescribed, and implemented to harness their benefits for youth.

#### Areas for Further Research

Previous reviews of TDIs, a broader category that goes beyond mobile interventions, have demonstrated the benefits of computer- and internet-based interventions, most commonly cognitive behavioral interventions, and most commonly examining the outcomes of depression, anxiety, and stress [51-58]. Similar to findings in adult populations, computer- and internet-based TDIs in youth samples have shown benefits, mostly in reducing internalizing symptoms, behavioral concerns, and eating disorders [51,52,54-60]. The literature on the efficacy of *mobile* TDIs, or mTDIs, is growing, with multiple meta-analytic reviews indicating positive impacts on a range of psychological outcomes in adults [42,53,61-63]. The emerging meta-analytic literature on mTDIs for *youth* is encouraging but limited, including generally beneficial results across (1) reviews blending a few trials of mTDIs together with mostly *nonmobile* TDIs in youth [52,64,65]; (2) a review combining 4 trials of mTDIs in children and adolescents with 21 trials of mTDIs in *adults* (mean age up to 59 years) [66]; (3) a recent meta-analysis of 12 youth trials, both with or *without* comparison groups, of smartphone apps exclusively on *internalizing* disorders [67]; and (4) another recent meta-analysis of 11 randomized controlled trials of smartphone apps for *depression*, *anxiety*, and *stress* in youth (aged 10-35 years) [68]. Although these initial findings are encouraging, a fitting next step for this emerging area of research is to meta-analytically review TDIs that are exclusively mobile in *youth* samples while including a *broad* array of youth clinical presentations and outcomes. Moreover, given the diverse designs of mTDIs for distinct youth characteristics and presenting problems, exploring the moderating influence of the interventions’ mobile technologies, theoretical orientations, technological and support features, and varying dosages would advance our ability to harness the full potential of mTDIs in improving youth well-being.

### The Current Meta-analysis: Goals and Hypotheses

The current 3-level meta-analysis evaluated the impact of wellness- and mental health-focused mTDIs (including smartphones and tablets, other types of mobile phones, and other handheld and wearable devices, including mobile VR) for youth, broadly defined as children, adolescents, and intentional (eg, university student) young adult samples or those with a mean age of ≤26 years. Improving upon some limitations of previous reviews, we included published and unpublished reports, only included controlled (either randomized or quasi-experimental) designs, and evaluated a broad range of participant clinical presentations (eg, unselected, at-risk, or clinical samples), intervention theoretical orientations, and outcomes. Drawing on evidence from prior reviews, we predicted that these mTDIs would yield significant benefits at postintervention on diverse indicators of youth well-being relative to comparison conditions. In addition, we examined the role of several potential moderators of intervention impact within the categories of methodological, youth, and intervention characteristics.

#### Methodological Characteristics

##### Timing of Outcomes

Prior reviews note that there are a limited number of studies assessing the long-term effects of TDIs [51,54] and mTDIs [68] on youth. The reviews that compare the effects at postintervention versus later follow-up periods have been mixed, with some finding that effects are stable into follow-up periods (eg, parenting TDIs [59]) and others finding that some or all effects diminish over time (eg, adult mTDIs [69] and parenting TDIs [70]). Given that youth might have added challenges in implementing long-term gains [57], we tentatively predicted that the timing of the outcome assessment (posttest vs follow-up) would moderate the strength of the mTDIs’ effects such that the effects of mTDIs would wane over time.

##### Outcome Type

Prior reviews have established the benefits of mTDIs in reducing depression, anxiety, and stress, mostly in adults [42,62,69], with emerging evidence in youth [67,68]. In addition, mTDIs have been shown to be effective in improving life satisfaction, quality of life, and psychological well-being [69]. To broadly evaluate the potential impact of mTDIs on youth, we examined a broad range of youth outcomes, including those that have not yet been examined in prior reviews. Therefore, we expected mTDIs to have a beneficial impact on depression, anxiety, stress, and well-being, and explored whether mTDIs would also have beneficial effects on other outcome types, such as psychosocial strategies and skills, interpersonal relationship factors, academic functioning, health-related behaviors, or knowledge.

##### Comparison Group Type

Reviews of TDIs and mTDIs have generally found that effects are largest when they are compared with no-intervention or wait-list groups and smaller when compared with groups that are more active and clinically potent [42,52,58,60,62,69-71]. Thus, we predicted that the comparison group type would moderate the effects of mTDIs. Specifically, we expected that mTDIs would demonstrate the strongest benefits compared with no intervention (eg, wait-list), followed by inert interventions (including information-only and attentional or placebo controls), and demonstrate noninferiority compared with clinical comparisons, including usual clinical care and established clinical interventions.

#### Youth Characteristics

##### Age

Several previous reviews of TDIs in youth have demonstrated that older participants experience a greater reduction in symptoms than their younger counterparts [51,55,65,72]; however, others have found no effect of age [57,59], and preliminary evidence on a small sample of mTDIs in adolescents and young adults also failed to find an effect of age [68]. In an exploratory fashion, we examined whether age moderates the effects of mTDIs.

##### Gender

The few reviews on the mental health benefits of smartphone apps that explored the role of participants’ gender have revealed nonsignificant effects in adult [42] and youth [59] samples. Nevertheless, given the differences in the rates of various mental health problems as a function of gender across development [73,74], we tested the effects of gender in an exploratory fashion.

##### Risk Level

Some prior reviews of mTDIs with adult samples have indicated that higher pretreatment severity (ie, clinical diagnosis or elevated mental health symptoms) is related to a greater reduction in symptoms and, therefore, produces a larger effect size (ES) [60,63]. In contrast, Pennant et al [54] found greater effects of computerized therapies for youth with subclinical symptoms versus a clinical diagnosis of anxiety; however, this effect was not found for depression. Two more recent reviews suggest inconclusive evidence regarding whether TDIs or mTDIs are more effective for youth who present with diagnoses or severe symptomatology [47,65]. Therefore, we examined participant risk level (ie, clinical diagnosis, elevated symptoms, and nonclinical sample) as an exploratory moderator.

#### Intervention Characteristics

##### Type of Technology

A prior review on the impact of mTDIs on both youth and adults did not find significant differences in effects by type of technology (ie, *smart* mobile phones/tablets vs other types of mobile phones, PDAs, wearable devices, or VR headsets) but noted that smartphone apps produced (nonsignificantly) larger ESs than both PDA and SMS text messaging interventions [66]. Given the limited information, we tested the impact of technology type as an exploratory moderator.

##### Guiding Theoretical Framework

Cognitive behavioral–based and mindfulness- or acceptance-based interventions are the most commonly examined theoretical frameworks among TDIs for youth and adults [51,54-58] and mTDIs for adults [42,53,62,69] and have generally yielded positive effects for problems such as anxiety, depression, externalizing behaviors, and quality of life. Thus, we hypothesized that mTDIs with cognitive behavioral–based and mindfulness- or acceptance-based theoretical frameworks would produce significant effects and examined the impact of additional theoretical orientations (eg, motivational or positive psychology) in a more exploratory fashion.

##### Technological and Support Features

Prior reviews have suggested that specific features of mTDIs, such as tailoring (ie, content shifting based on responses), gamification, and automatic reminders, may increase engagement and yield more robust effects [53,75]; however, much of this research has been conducted with adult samples. Therefore, we explored the potential moderating impact of various technological features such as personalization, tailoring, gamification, and social elements (eg, peer mentoring).

Research also points to the possibility that support from a human or virtual (“bot”) professional, who can provide guidance, coaching, accountability, and, in some cases, supervised skills practice, may lead to increased adherence to mTDIs [47,76,77]. Indeed, several reviews have indicated that self-guided interventions are generally more effective with some access to human support or guidance, in part because of increased engagement and adherence [53,78,79]. Similarly, research on psychotherapy and other in-person youth interventions have highlighted the benefits of supervised practice in contributing to youths’ psychological skill development, especially when delivered over multiple sessions [79-84]. Nevertheless, the overall evidence on human support for mTDI use is mixed, with a handful of reviews *not* finding added benefits for interventions incorporating human support as compared with those that do not [42,54,55,85]. Thus, we explored the potential impact of human or bot support elements, such as coaching, supportive accountability, and supervised skills practice.

##### Dosage: Frequency and Duration

Previous studies have rarely investigated the dose-response relationship between TDI use and outcomes, and those that have done so tend to yield mixed results [47,51,54]. Some reviews have found that higher dosages, or longer durations, predicted a greater reduction in symptoms with particular outcomes (eg, problem behavior or depression) [59,86]. However, other studies were unable to establish such a relationship [57,85]. Therefore, we explored whether the prescribed or completed frequency (eg, weekly) or duration (eg, minutes, sessions, or weeks) of the intervention moderated the benefits of mTDIs.

## Methods

### Search Strategy and Study Selection

We used 5 systematic search strategies to assemble an unbiased, representative sample of published and unpublished controlled trials. First, we conducted searches for reports appearing through March 2021 in 5 academic databases: PsycINFO, ERIC, ProQuest Digital Dissertations, MEDLINE (Web of Science), and PubMed. We used a combination of several groups of search terms to find studies meeting our criteria for (1) participants (eg, child*, adolescen*, *teen*, *youth*, *young adult*, *university student*), (2) interventions (eg, *mental health*, *psychological*, *intervention*, *cognitive behavioral*, *mindfulness*), (3) mental health (eg, depress*, anxiety*, *well-being*), (4) technology (eg, smartphone app*, mobile app*, *tablet-based*), and (5) research design (eg, *RCT*, *controlled trial*, *clinical trial*, *quasi*, *comparison group*, *PRISMA*). Second, we also inspected the reference lists of each study meeting our criteria and of relevant previous reviews. Third, we hand-searched the contents of 16 selected journals most likely to publish studies on mobile mental health interventions involving youth. Fourth, we hand-searched the contents of proceedings for the recent years of 7 relevant academic conferences. Finally, we contacted authors of prior reviews, reports, and conference proceedings relevant to our sample to inquire about additional published or unpublished evaluations of fitting trials. Further details on these search strategies are provided in Multimedia Appendix 1.

To be included in our final sample, the studies had to meet six criteria: (1) examine an *automated* psychological or behavioral intervention, either selecting participants based on a diagnosis or risk factor or targeting an unselected sample to promote mental health and wellness; (2) deliver the intervention primarily via *mobile* (handheld or wearable) technology, including pre–cellular technology handheld computers (eg, palm pilot and PDA), mobile cellular phones or tablets (eg, iPad and iPod touch)—using SMS text messaging, instant messaging, or more current mobile mental health apps—and wearable devices (eg, smart watch, smart glasses, VR headsets that are fully self-contained or linked with a mental health app on a smartphone or tablet device that is portable and able to be used in the participant’s home); (3) contain at least one quantitatively assessed mental or behavioral health *outcome* measure (described in the following sections) for which ESs could be calculated; (4) target *youth*, broadly defined as children, adolescents, and intentional (eg, university student) young adult samples or those with a mean age ≤26 years (including interventions delivered to parents that targeted youth outcomes); (5) include a *comparison group* with at least 10 participants assigned to each condition; and (6) be reported in English, Spanish, Dutch, or German.

We excluded interventions with a primary focus on academics or physical health (eg, nutrition, weight loss, or diabetes management) but included studies that focused on psychobehavioral health such as smoking cessation, insomnia, and disordered eating. We did not include interventions delivered through audio or video tapes or videodiscs, a local computer program, or a website only. In addition, we did not include mobile interventions that were primarily reliant on human support (eg, therapists sending messages). Finally, we excluded interventions comprising solely medication reminders.

**Figure 1:**
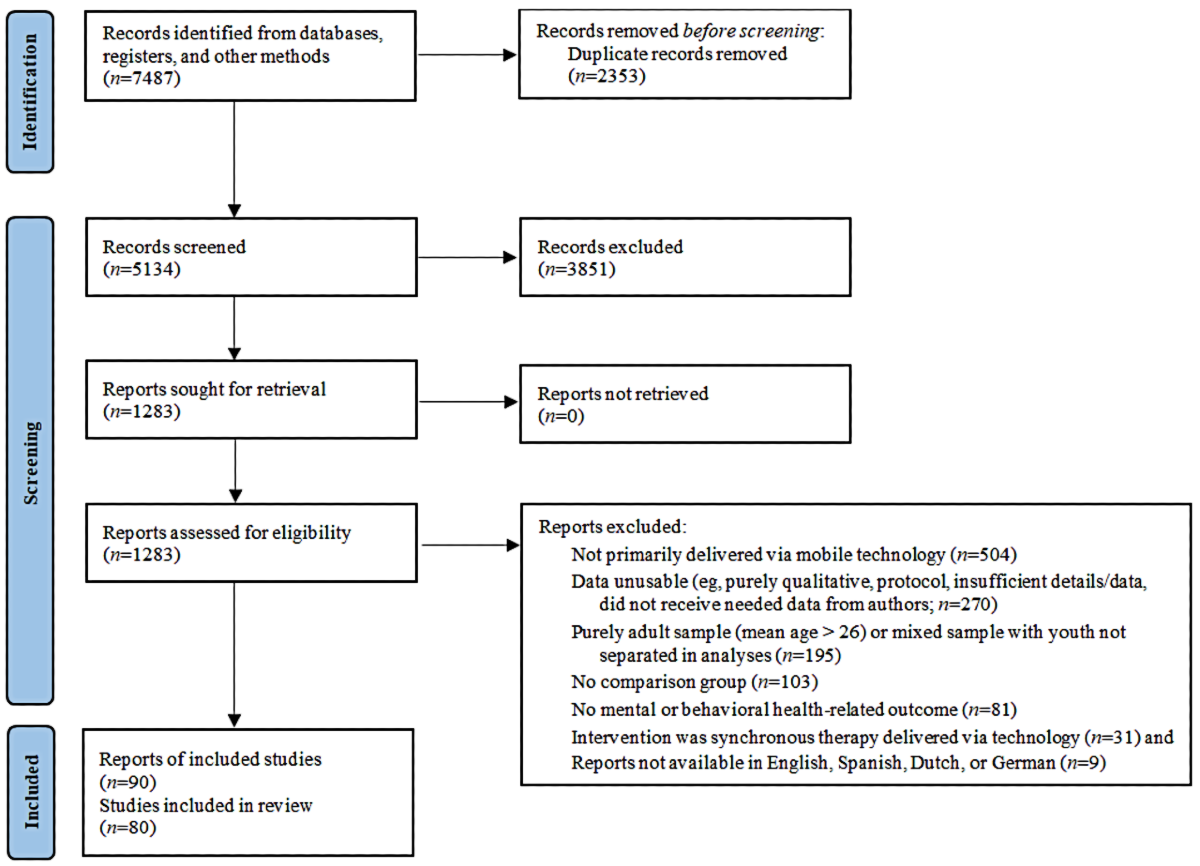
PRISMA (Preferred Reporting Items for Systematic Reviews and Meta-Analyses) flow diagram of the study selection process.

Figure 1 shows a PRISMA (Preferred Reporting Items for Systematic Reviews and Meta-Analyses) flow diagram of sample searching, selection, and inclusion. The aforementioned search procedures identified 7487 potentially relevant reports, including 2353 (31.43%) duplicates that were removed. An additional 51.44% (3851/7487) of reports were eliminated as they did not meet our inclusion criteria. Among the 1283 eligible reports, some contained variants of the same intervention (eg, 2 interventions with the same active component but varying lengths), and we only included the intervention that was more comprehensive (ie, contained more elements or was longer in duration) or completely technology-based. However, if conceptually distinct interventions (eg, 2 different apps using different techniques) were evaluated in the same report, each intervention was coded separately. Data from multiple reports on the same sample and intervention were combined into a single report, reducing 16 overlapping reports to a sample size of 6.

In cases where means and SDs were not included in the original reports or effects could not be calculated because of insufficient data, we attempted to contact study authors to secure missing data. On the basis of a lack of author response, we excluded 16 studies for which no ESs could be calculated for any relevant outcome measure. This screening process led to a final sample of 83 interventions reported in 80 studies between 2005 and 2021.

### Study Coding (Data Extraction)

#### Methodological Characteristics

For each report, we coded the year of the report, publication status, country in which the intervention took place, type of experimental design and comparison group, sample size, outcome types, and additional codes described in the following sections.

##### Timing of Outcomes

We coded the number of weeks between pre- and postintervention outcome assessments and between postintervention and each follow-up assessment period.

##### Outcome Type

We coded a broad array of outcomes to capture the various psychosocial and related aspects of functioning that might be affected by mTDIs. The relevant outcomes assessed in our sample of studies were classified into 14 possible categories, some of which were conceptually nested under higher-order categories, as noted in Textbox 1.

**Outcome types coded.**

- General psychological well-being or distress included 2 subcategories:
  ***Stress*** (eg, perceived and physiological indices of stress)
  ***General or global psychological distress*** and well-being (eg, distress, positive and negative affect, mood states, quality of life, happiness, or life satisfaction)
- Psychosocial strategies or skills included 2 subcategories:
  ***Social-cognitive strategies or styles*** (eg, different types of affective, cognitive, and social skills related to effective coping strategies, help-seeking behaviors, or mindfulness practices; overcoming dysfunctional beliefs, rumination, or hostility; resilience; or emotional self-awareness and regulation)
  ***Self-perceptions*** (eg, self-esteem or self-efficacy)
- Internalizing symptoms included 2 subcategories:
***Depression***
***Anxiety***
- ***Other (noninternalizing) mental health problems*** (eg, autism, attention deficit hyperactivity disorder, or eating disorders)
- ***Health and health behavior*** (eg, substance use, sleep, physical activity, pain, or eating behaviors)
- ***Interpersonal relationships*** (eg, conflict, perceived social support, belongingness, loneliness, or social skills)
- ***Academics*** (eg, academic performance or adjustment)
- ***Psychology or health-related knowledge*** (eg, knowledge about topics such as substance use norms and consequences or sleep hygiene)
- ***Psychosocial outcomes in someone other than the target youth*** (eg, parent stress, warmth, use of punishment)
- ***Other*** (eg, perceptions of productivity, stigma, or close friend’s smoking behavior)
- ***Intervention (ie, app) ratings*** (eg, intervention feasibility and social validity, acceptability of the mobile technology–delivered intervention (mTDI), and its uptake or use)

##### Comparison Group Type

Studies were coded as having 1 of 3 different comparison groups. The majority of studies included a *no-intervention* (eg, wait-list control) condition in which the comparison group only completed assessment procedures. Some studies compared the intervention of interest with an *inert* comparison group, whether information-only (eg, pamphlets or website links to general health-related information), attention-placebo (eg, passive SMS text messages), or minimal treatment-as-usual (eg, standard protocol before a medical procedure) conditions that did not contain the therapeutic elements of the evaluated intervention. These comparison groups generally attempted to control for nonspecific factors such as attention or social interaction. Finally, some studies included a *clinical* comparison group, whether a usual clinical care comparison or some other established (validated or otherwise intended to be beneficial) intervention. In some studies with a clinical comparison group, both the mTDI and comparison groups received a similar base intervention (eg, counseling vs counseling+app) and thus tested the *added* or incremental benefit of the mTDI of interest.

#### Youth Characteristics

##### Age, Gender, Race, and Ethnicity

When the information was available, studies were coded for the sample age (mean, SD, and range), gender, race, and ethnicity.

##### Risk Level and Type

We coded whether researchers selected participants based on particular symptoms or risk factors into the following categories: (1) *psychological clinical sample* (ie, symptoms indicative of a Diagnostic and Statistical Manual of Mental Disorders diagnosis) [87,88], (2) *psychological or mental health at-risk sample* (ie, subclinical symptoms of psychopathology), (3) *nonmental health risk* (ie, medical risk, diagnosis, or procedure), and (4) *general (unselected) community sample* not selected for any particular risk factor.

#### Intervention Characteristics

##### Type of Technology

We coded each intervention’s primary and secondary (if relevant) type of technology into one of the following categories: (1) *smartphone or tablet* (eg, iPhone, iPad, or iPod touch), (2) *presmartphone mobile device* (eg, presmart mobile phone, palm pilot, or PDA), (3) *mobile VR* (eg, headsets) or *video game* (ie, handheld), and (4) *other wearable devices* (eg, smart watch, biosensor or activity monitor, and smart glasses). VR headsets and other wearable devices were typically used in conjunction with smartphones or tablets. Finally, some interventions were also able to be accessed on a (5) *computer* as a *secondary* type of technology.

##### Guiding Theoretical Framework

Interventions were coded as having one of the following primary guiding theoretical frameworks: (1) *cognitive behavioral*, (2) *mindfulness- or acceptance-based* (eg, mindfulness-based stress reduction, mindfulness-based cognitive therapy, acceptance and commitment therapy, or dialectical behavior therapy), (3) *blended cognitive behavioral and mindfulness*, (4) *other or multiple* (ie, positive psychology, interpersonal, motivational or stages of change, and transtheoretical), or (5) *atheoretical or not specified*. When mTDIs were available in the commercial market, they were consulted directly to supplement the information obtained from the research reports.

##### Technological and Support Features

We coded whether the intervention included *personalization* (ie, the ability to alter the app environment through features such as personal preferences; personal dashboards; or use of photos, music, or contacts), *tailoring* (ie, the use of algorithms that alter intervention content based on contact sensing, prior responses, feedback, or other input), a *social component* (eg, forum or social media use or mentoring), or *gamification* (eg, rewards, badges, points, levels, or quests).

We also coded several intervention features designed to support participants in using the mTDI: (1) *training or orientation* for the participants about using the mTDI (eg, virtual training within the app or via email or video chat, in-person training, or a paper manual); (2) *in-person element* besides orientation or training (eg, simultaneous counseling); (3) *reminders* sent to encourage the use of the mTDI, either automatically through the app (eg, push or banner notifications) or outside of the app (eg, emails, texts, or calendar reminders); (4) *human or bot (automatic) support* (eg, supportive SMS text messages, phone calls, or personalized feedback) specifically around the mTDI; (5) targeted guidance indicative of *supportive accountability*, designed to increase adherence to an intervention via support and accountability from a trustworthy coach who assists with setting process-oriented expectations and goals [77]; and (6) targeted guidance in the form of *supervised skills practice* [79].

##### Dosage: Frequency and Duration

When the information was available, studies were coded for the intervention’s prescribed and completed (both objectively determined and self-reported) frequency and duration. Specifically, the frequency of use was coded as one of the following categories: at least 4 days per week or as much as feasible, 2 to 3 days per week, once per week, less than once per week, one-time session, and not stated or at user discretion. The intervention duration was coded in terms of minutes, weeks, and sessions. When data were available, we also calculated the percentage of the completed duration of the intervention by dividing the completed duration by the prescribed duration.

### Risk of Bias (Quality) Assessment

For study quality, we followed the approach of an integrative study quality coding scheme [89] designed to draw upon the strengths of several previously validated quality indices, including the Cochrane Collaboration’s tool for assessing the risk of bias [90-92]. This coding scheme rates each study on 10 features: peer review and impact factor, experimental design, sample size, attrition, reliability of measures, validity of measures, adjustment for pretest differences, intent-to-treat analysis, reporting of sample characteristics, and involvement of study authors in mTDI development. Each feature is rated on a 4-point scale (from 0, indicating the lowest quality, to 3, indicating the highest quality). The 10 item scores are then summed, resulting in a score for which a score of 20 represents average or normal research practices.

### Reliability of Coding

A team of 5 trained postbaccalaureate and graduate students assessed the studies for eligibility and inclusion in the meta-analysis and met weekly to review any questions for consensus. A team of 6 graduate students with advanced clinical and quantitative training then reviewed and coded eligible reports for descriptive features, moderators, quality indicators, and outcome data. The coders were supervised by 3 faculty members with expertise in clinical psychology, mTDIs, and meta-analytic procedures. After the iterative training phase, coders had ongoing opportunities for consensus checks through a consultation system and weekly faculty supervision. From a subsample of 18 to 31 studies (depending on the code) containing 44 interventions, 46 comparisons, and 108 ESs, any code that did not reach adequate reliability (ie, >0.80 κ, 85% agreement, or 0.95 intraclass correlation coefficient, as fitting) [93,94] was reviewed by at least one other coder in the entire sample. Lead authors provided an additional review of randomly selected articles throughout the coding process. Any questions or discrepancies were resolved through discussions.

### Meta-analytic Strategy

#### ES Calculation

Cohen *d* was calculated for each outcome to reflect the effect of mTDIs relative to the comparison condition, with positive ESs representing outcomes in which the intervention group outperformed the comparison group. If *d* values could not be obtained directly from primary studies, the formulas by Borenstein et al [95] and Lipsey and Wilson [96] were used to transform the reported statistical information into Cohen *d*. Whenever possible, *d* values were calculated using means and SDs, frequencies or proportions, odds ratios, or results from *F* or *t* tests. If a primary study did not report sufficient information to extract or calculate the ES, the study authors were contacted for additional information. When the only information available indicated that an ES was nonsignificant, we conservatively set that ES to zero, following Mullen [97]. This procedure was preferred above excluding primary studies from the review, as the latter would reduce the statistical power in the analyses. To correct for pretreatment differences, we adjusted the postintervention and follow-up effects for preintervention baseline outcome levels (using subtraction, similar to procedures in other meta-analyses) [89,98,99] when pretreatment data were available. Finally, before analysis, all ESs were converted to Hedges *g* to account for potential bias in small sample sizes.

#### The 3-Level Meta-analytic Model

Most primary studies included in this review reported on multiple intervention effects, typically because multiple outcomes were tested or multiple comparison conditions were part of the study design. The resulting dependency in ESs (ie, the fact that ESs extracted from the same study are more alike than the ESs extracted from different studies) violates the assumption of independent ESs underlying traditional meta-analytic techniques [96].

Therefore, a 3-level random-effects model was used for all analyses [100-104]. In this 3-level model, 3 sources of variance were modeled: sampling variance of the observed ESs (ie, sampling variance; level 1), variance between ESs derived from the same study (ie, within-study variance; level 2), and variance in ESs derived from different studies (ie, between-study variance; level 3). The sampling variance at level 1 of the model is not estimated but considered known and calculated using the formula given by Cheung [101].

To determine whether testing select moderators would be informative, we first examined the ES heterogeneity by testing the significance of the within-study variance (level 2) and the between-study variance (level 3). We performed 2 one-sided log-likelihood ratio tests in which the deviance of the full model was compared with the deviance of the model without one of these variance parameters. If the within-study variance or the between-study variance were significant, we proceeded with the moderator analyses. The coded variables were only tested as moderators when (categories of) these variables were based on at least three studies or three ESs. In some cases, we consolidated categories with <3 studies or ESs into another (or the *other*) category.

#### Software and Parameters

We used the function *rma.mv* of the *metafor* package [105] in the R statistical environment (version 3.6.1; R Foundation for Statistical Computing) [106], following the setup and R syntax by Assink and Wibbelink [100], to model the 3 sources of ES variance [103,104]. The overall effect was estimated using an intercept-only model, and potential moderators were examined by adding these variables as covariates to the intercept-only model. The *t* distribution was used in testing individual regression coefficients of the models and for calculating the corresponding CIs [107]. When models were extended with categorical moderators comprising >3 categories, the omnibus test followed an *F* distribution. The restricted maximum likelihood estimation method was used to estimate the model parameters. Before conducting moderator analyses, continuous variables were centered on their means, and dichotomous dummy variables were created for categorical variables. The log-likelihood ratio tests were conducted as 1-tailed, whereas all other significance tests were conducted as 2-tailed. The significance level was set to 0.05 in all analyses, and 95% CIs were estimated.

#### Publication Bias

A problem that may arise in meta-analysis is the *file drawer problem* [108], in that studies with nonsignificant or negative results are less likely to be published than studies that produced significant and positive results. To reduce this problem, we attempted to be exhaustive in our search strategy to retrieve both published and unpublished primary studies. To further assess bias in our data set of ESs, 2 analyses were conducted. First, we performed the trim-and-fill analysis by Duval and Tweedie [109,110] to examine the symmetry of a funnel plot in which ESs were plotted against their SEs. In the case of publication bias, the plot is asymmetrical, as the ESs are missing to the left of the estimated mean. The trim-and-fill algorithm estimates these *missing* ESs using an iterative nonparametric method. After imputing these ESs, the symmetry of the plot is restored, and an *adjusted* overall effect can be estimated. We also examined bias by performing the Egger test, in which ESs are regressed on their SEs [111]. This was performed by adding the SE as a covariate to an intercept-only, 3-level meta-analytic model. In this model, a significant positive slope indicated the presence of publication bias.

## Results

### Study Sample and Descriptive Characteristics

Multimedia Appendix 2 [112-200] provides a table with details about each of the 80 studies eligible for this meta-analysis, 3 (4%) of which contained 2 eligible interventions and 10 (13%) that contained 2 eligible comparison groups, yielding 83 interventions, 93 comparisons, and a combined sample size of 19,748 youth. Of these 80 studies, 76 (95%) provided estimates of 484 postintervention ESs, and 29 (36%) studies provided estimates of 225 follow-up ESs.

Several aspects of the 80 included studies are worthy of comment. First, most (68/80, 85%) of the studies were published in peer-reviewed scientific journals, and the remainder were unpublished dissertations or in-preparation manuscripts that extended prior peer-reviewed work published as a pilot trial or presented at an academic conference. In addition, most of the studies were published within the past decade or so, with 96% (77/80) published since 2010 and 28% (22/80) since 2020. Of the 80 studies, 33 (41%) were conducted in the United States, with 36 (45%) reports from the broader North American continent, 23 (29%) from Europe, 11 (14%) from Australia, 9 (11%) from Asia or the Middle East, and 1 (1%) from South America.

In terms of participants, across the 93% (74/80) of studies reporting relevant demographic information (and among the 67/80, 84% of studies reporting SD), the average age ranged from 2.93 to 26.25 (weighted mean 15.92; SD 2.86) years, and on average, 63.83% of study samples were female (but notably, most studies did not report on, or likely assess, gender other than female or male). Only 38% (30/80) of studies provided a full breakdown of participant race and ethnicity, and 23% (18/80) provided no information on these demographics at all. Furthermore, 63% (50/80) of studies selected participants based on one or more risk factors versus recruiting a general community sample. The most common risk factor used for participant recruitment and screening was subclinical psychological risk (eg, substance use or elevated depression; 30/80, 38% of studies), followed by some nonmental health risk (12/80, 15% of studies; in all cases within this sample, this was a medical diagnosis such as spina bifida or obesity or a medical procedure such as surgery or dental work), and, finally, participants with a clinical psychological or psychiatric diagnosis (eg, anxiety or autism; 8/80, 10% of studies).

In terms of the 83 interventions, 74 (89%) used smartphones or tablets (1 used an iPod touch); 4 (5%) used presmartphone mobile devices (all phones, including Motorola A925, Sony Ericsson, and Vodafone); 4 (5%) used VR headsets, either freestanding or in conjunction with a mobile phone app; and 1 (1%) used a handheld video game. Most (70/83, 84%) of the interventions took place in participants’ daily environments; however, several (13/83, 16%) took place in a medical setting (eg, to address anxiety or pain related to a medical procedure). The most prevalent guiding theoretical framework of the mTDIs was cognitive behavioral (36/83, 43% of the interventions), followed by other or transtheoretical frameworks (eg, positive psychology and motivational; 20/83, 24%), mindfulness- or acceptance-based (17/83, 20%), and a few atheoretical or unspecified frameworks (3/83, 4%). Furthermore, in nonexclusive categories, the interventions’ technological features included personalization (18/83, 22%), tailoring (36/83, 43%), a social component (10/83, 12%), and gamification (20/83, 24%). In terms of support features, of the 83 interventions, 30 (36%) included some sort of orientation or training (either virtual or in person), 12 (14%) contained one or more other in-person element, 40 (48%) incorporated reminders to encourage the use of the intervention, and 22 (27%) included some form of human or bot support or guidance, with 20 (24%) containing supportive accountability and 9 (11%) containing supervised skills practice.

All (80/80, 100%) studies provided some information about the prescribed or completed dosage (or both) of their interventions, whether objectively pulled from the mTDI or self-reported by the participants; however, the specific details reported were variable. Of the 83 interventions, 13 (16%) were single-session interventions and the remainder were prescribed to range from 4 to 2505 sessions (weighted mean 89.86, SD 374.29; *k*=42 studies reporting on 44 interventions) across a time span of 2 days to 43.45 weeks (weighted mean 7.48, SD 7.46; *k*=65 studies reporting on 68 interventions). Of these 70 interventions (contained in 67 studies), 43 (61%) were prescribed for daily use, 9 (13%) for 2 to 3 days per week, 7 (10%) for once a week, and the remaining 11 (16%) were either prescribed to be used as needed or at the user’s discretion or not stated in the report. In terms of duration of use, the prescribed minutes of use for interventions ranged from 5 to 3650 minutes (mean 345.25, SD 789.95; *k*=32 studies reporting on 32 interventions). Notably, only 47 out of 80 (59%) studies provided some sort of objective information about how much participants *actually* engaged in the intervention (eg, number of sessions, minutes, or weeks). Using all available information, we calculated the intervention *completion* percentage and found the average to be 85.05% of the researchers’ prescribed sessions (*k*=29 studies reporting on 30 interventions), 87.19% of the prescribed intervention minutes (*k*=13 studies reporting on 13 interventions), and 86.7% of the prescribed intervention weeks (*k*=37 studies reporting on 43 interventions).

Studies assessed a variety of psychosocial outcomes, which we originally coded in 14 categories (see the *Methods* section) and then consolidated into 6 categories because of conceptually similar content or small numbers of studies or effects (see the final list of consolidated categories in the note below Multimedia Appendix 2).

Notably, 25% (20/80) of studies also provided information about the intervention group’s ratings on measures of the intervention’s social validity (eg, user satisfaction, perceived usefulness, quality, usability, or acceptability). As these data were generally only available at the posttest time points and for intervention but not comparison groups, we do not report ESs on these types of outcomes. However, to analyze trends in diverse measures of mTDI social validity across all studies with such data available, we standardized all available Likert scale ratings for these constructs onto a single scale, with 0 representing the lowest and 100 the highest possible rating of social validity. On this standardized scale, the average rating (weighted by included sample size) for self-report scores of the mTDIs’ social validity was 58.24 and ranged quite widely across studies (30.20-100).

There was variability in the types of comparison groups as well. Slightly less than half (37/80, 46%) of the comparisons involved groups such as wait-lists that contained no active intervention, whereas the remainder of the comparisons involved either passive information-only or placebo groups (28/80, 35%) or, less commonly, clinical comparisons that were intended to have therapeutic benefits (15/80, 19%). For the studies (43/80, 54%) that used an active (inert or clinical) comparison, the modality of the comparison group was distributed fairly evenly across in-person (20/43, 47%) and other technology-based interventions (18/43, 42%), with just a few (5/43, 12%) having some other modality (ie, blended interventions containing both technology and in-person elements or paper-and-pencil materials).

### Average Effect of mTDIs

The average ES across all possible comparisons within the 80 studies (yielding 709 ESs across posttest and follow-up assessments) was *g*=0.27 (*P*<.001; 95% CI 0.20-0.33). There was significant heterogeneity across studies (*σ*^2^ level 3=0.06; *P*<.001; 51.76% of the variance among ESs), as well as between ESs extracted from the same study (*σ*^2^ level 2=0.03; *P*<.001; 27.50% of the variance among ESs). Random sampling error accounted for 20.74% of the variance. To explore the substantial variability between and within studies, a number of moderators were considered. These analyses are described in the following 3 sections and detailed in Table 1.

**Table 1:** Moderators of the effectiveness of mobile technology–delivered interventions for youth^a^.

Characteristics						*k^b^*			Effect sizes, n			B_0_ (intercept), *g* (95% CI)			B_1_ (slope), *g* (95% CI)			*F* (df_1_, df_2_)					*P* value		
**Methodological characteristics**																									
	Study quality					80			709			0.25 (0.19 to 0.31)^***^			–0.02 (–0.04 to –0.01)^**^			7.03 (1, 707)					.01^c^		
	**Timing of outcome**																			0.07 (1, 707)					.79
		Posttest (RC^d^)			76			484			0.27 (0.21 to 0.33)^***^			N/A^e^											
		Follow-up			29			225			0.26 (0.19 to 0.34)^***^			–0.01 (–0.06 to 0.05)											
	**Outcome type**																			2.70 (5, 703)					.02
		General psychological well-being or distress (RC)			35			98			0.28 (0.20 to 0.37)^***^			N/A											
		Internalizing (depression, anxiety)			44			145			0.30 (0.22 to 0.39)^***^			0.02 (–0.06 to 0.10)											
		Other (noninternalizing) mental health			7			42			0.21 (0.04 to 0.38)^*^			–0.07 (–0.25 to 0.10)											
		Psychosocial strategies and skills			26			161			0.34 (0.25 to 0.42)^***^			0.05 (–0.02 to 0.13)											
		Health (behavior; eg, substance use)			35			190			0.24 (0.16 to 0.32)^***^			–0.04 (–0.15 to 0.06)											
		Other (eg, knowledge or relationships)			20			73			0.15 (0.04 to 0.25)^**^			–0.14 (–0.25 to –0.03)^**^											
	**Comparison group type**																			0.17 (2, 706)					.84^c^
		No intervention (RC; eg, wait-list)			41			376			0.28 (0.20 to 0.36)^***^			N/A											
		Inert (eg, placebo or information-only)			30			186			0.26 (0.18 to 0.35)^***^			–0.02 (–0.11 to 0.08)											
		Clinical (eg, established intervention)			18			147			0.24 (0.12 to 0.36)^***^			–0.04 (–0.17 to 0.09)											
	Mean age (years)					74			669			0.26 (0.19 to 0.32)^***^			0.003 (–0.01 to 0.01)			0.17 (1, 667)					.68^c^		
	Gender (percentage female)					77			699			0.26 (0.20 to 0.33)^***^			0.002 (–0.002 to 0.01)			1.06 (1, 697)					.30		
	**Risk level and type**																			3.15 (3, 705)					.02^c^
		General sample not selected for risk (RC)			30			247			0.19 (0.09 to 0.29)^***^			N/A											
		Nonmental health (ie, medical) risks			12			46			0.52 (0.33 to 0.72)^***^			0.33 (0.11 to 0.55)^**^											
		Psychological or mental health at-risk sample			30			332			0.29 (0.19 to 0.39)^***^			0.09 (–0.05 to 0.23)											
		Psychological clinical sample (diagnosis)			8			84			0.20 (0.01 to 0.39)^*^			0.01 (–0.21 to 0.22)											
**Intervention characteristics**																									
	**Primary type of technology**																			0.82 (2, 706)					.44
		Smartphone or tablet (RC)			71			665			0.25 (0.19 to 0.32)^***^			N/A											
		Presmartphone mobile device			4			23			0.37 (0.09 to 0.65)^*^			0.11 (–0.18 to 0.40)											
		Mobile VR^f^ headset or handheld video game			5			21			0.41 (0.13 to 0.68)^**^			0.15 (–0.13 to 0.44)											
	**Guiding theoretical framework**																			1.04 (4, 704)					.38
		Cognitive or behavioral (RC)			35			249			0.31 (0.21 to 0.40)^***^			N/A											
		Mindfulness or acceptance			16			238			0.28 (0.15 to 0.42)^***^			–0.02 (–0.19 to 0.15)											
		Cognitive behavioral and mindfulness			6			46			0.35 (0.12 to 0.58)^**^			0.05 (–0.02 to 0.29)											
		Other or multiple (eg, motivational)			20			171			0.16 (0.04 to 0.29)^**^			–0.14 (–0.30 to 0.01)^g^											
		Atheoretical or not specified			3			5			0.41 (–0.04 to 0.85)^g^			0.10 (–0.35 to 0.56)											
	**Intervention technological features**																								
		**Personalization**																	1.37 (1, 706)					.24	
			Absent (RC)	63			575			0.28 (0.21 to 0.35)^***^			N/A												
			Present	17			133			0.19 (0.07 to 0.32)^**^			–0.09 (–0.23 to 0.06)												
		**Tailoring**																	0.84 (1, 706)					.36	
			Absent (RC)	45			399			0.28 (0.20 to 0.37)^***^			N/A												
			Present	34			309			0.23 (0.13 to 0.32)^***^			–0.06 (–0.18 to 0.07)												
		**Social component**																	0.17 (1, 706)					.68	
			Absent (RC)	70			628			0.26 (0.20 to 0.33)^***^			N/A												
			Present	9			80			0.22 (0.05 to 0.40)^*^			–0.04 (–0.23 to 0.15)												
		**Gamification**																	0.78 (1, 706)					.38	
			Absent (RC)	60			531			0.24 (0.17 to 0.31)^***^			N/A												
			Present	19			177			0.31 (0.18 to 0.44)^***^			0.07 (–0.08 to 0.21)												
	**Intervention support features**																								
		**Orientation to or training on mTDI^h^**																	0.09 (1, 678)					.77	
			Absent (RC)	48			361			0.27 (0.19 to 0.35)^***^			N/A												
			Present	29			319			0.25 (0.15 to 0.35)^***^			–0.02 (–0.15 to 0.11)												
		**Other in-person element**																	0.004 (1, 707)					.95	
			Absent (RC)	68			637			0.27 (0.20 to 0.33)^***^			N/A												
			Present	12			72			0.27 (0.11 to 0.43)^***^			0.006 (–0.16 to 0.17)												
		**Reminders**																	1.91 (1, 696)					.17	
			Absent (RC)	40			233			0.31 (0.21 to 0.40)^***^			N/A												
			Present	37			465			0.22 (0.13 to 0.30)^***^			–0.09 (–0.22 to 0.04)												
		**Guidance, coaching, and feedback**																	0.24 (1, 707)					.62	
			Absent (RC)	59			531			0.28 (0.20 to 0.35)^***^			N/A												
			Present	21			178			0.24 (0.12 to 0.36)^***^			–0.04 (–0.18 to 0.11)												
		**Supportive accountability**																	0.14 (1, 707)					.71	
			Absent (RC)	62			538			0.27 (0.20 to 0.35)^***^			N/A												
			Present	18			171			0.25 (0.12 to 0.38)^***^			–0.03 (–0.18 to 0.12)												
		**Supervised skills practice**																	0.62 (1, 707)					.43	
			Absent (RC)	72			651			0.26 (0.19 to 0.33)^***^			N/A												
			Present	8			58			0.34 (0.14 to 0.54)^***^			0.08 (–0.13 to 0.30)												
	**Dosage: prescribed frequency of use**																			4.39 (4, 704)					.002^c^
		As much as feasible; ≥4 days per week (RC)			43			507			0.23 (0.15 to 0.30)^***^			N/A											
		Some days or more than once a week			9			53			0.27 (0.09 to 0.44)^***^			0.04 (–0.15 to 0.23)											
		About once a week			7			58			0.53 (0.33 to 0.73)^***^			0.30 (0.09 to 0.52)^**^											
		One-time session			13			45			0.46 (0.28 to 0.63)^***^			0.23 (0.04 to 0.42)^*^											
		Not stated, when needed, or at user discretion			8			46			0.08 (–0.09 to 0.24)			–0.15 (–0.33 to 0.03)											
	**Dosage: prescribed duration of intervention**																								
		Weeks			78			697			0.27 (0.21 to 0.34)^***^			–0.003 (–0.01 to 0.01)			0.35 (1, 695)					.56			
		Sessions			54			414			0.33 (0.24 to 0.41)^***^			–0.0001 (–0.0003 to 0.0002)			0.44 (1, 412)					.51			

^a^The right columns list the omnibus *F* test and *P* value for each moderation test. The middle columns list the intercept (B_0_), or mean effect size, and slope (B_1_), an estimated unstandardized regression coefficient, of the relevant Hedges *g* statistics, with CIs around each. Effects and slopes that differ significantly from 0 are denoted with asterisks in the intercept (B_0_) and slope (B_1_) columns, respectively. For categorical moderators, each intercept represents the mean effect of a category, whereas each slope represents the difference in the mean effect between the category and reference category. Depending on its sign, the slope of a continuous moderator represents an increase or decrease in the effect size with each unit increase in the variable.

^b^Number of studies with relevant effect size data for a given row. In cases of multiple interventions or comparisons, some studies were counted in multiple rows; thus, these numbers sometimes exceeded 80. Owing to missing data, some counts fall short of 80. Further details on what was included in different categories of the included moderators are provided in the *Methods* section.

^c^Significance of moderation analysis changed when conducted on a subsample of the highest-quality studies (*k*=38; Table 2).

^d^RC: reference category.

^e^N/A: not applicable (as the slope represents a comparison with the reference category).

^f^VR: virtual reality.

^g^*P*<.10.

^h^mTDI: mobile technology–delivered intervention.

*P<.05.

**P<.01.

***P<.001.

### Differences in Effects of mTDIs Based on Methodological Characteristics

#### Study Quality and Publication Bias

**Figure 2:**
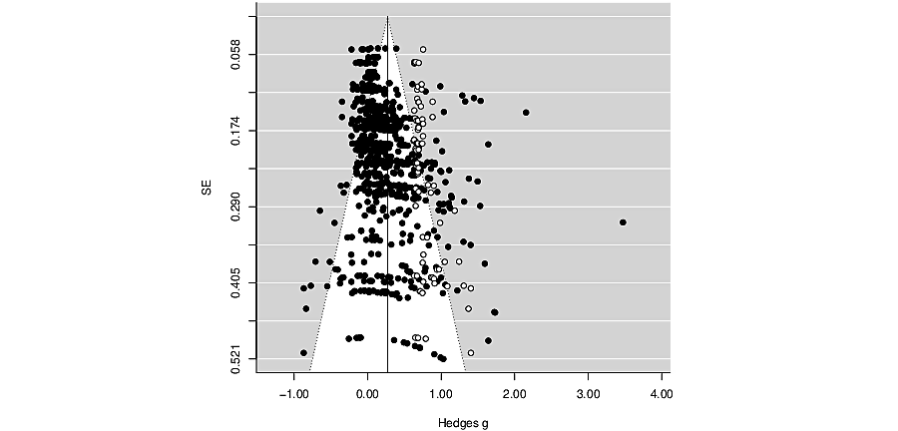
Funnel plot of observed mTDI effects (solid circles) and imputed effects (open circles) plotted against their standard error. mTDI: mobile technology–delivered intervention.

Overall study quality significantly moderated the overall effect of mTDIs in such a way that ESs decreased as the quality index increased (Table 1). The slope indicated that for every 1-point increase in quality index score, the effect decreased by 0.02. Given this moderation effect, we also ran all analyses with only the higher-quality studies—that is, studies that achieved a total quality index >20, which denotes studies that, on average, surpassed benchmarks for average-quality research methods [89]. Unless otherwise noted in the relevant presentation of results in the following sections, the pattern and significance of the results with this reduced, higher-quality sample of studies were identical to those of the full sample of studies. However, in cases where the statistical significance of results shifted when tested with only higher-quality studies, the results from analyses with only the higher-quality studies are presented separately in Table 2 (full set of results available from authors upon request). The average ES across all possible comparisons within the 38 higher-quality studies (yielding 428 ESs across posttest and follow-up assessments) was *g*=0.20 (*P*<.001; 95% CI 0.13-0.27). A funnel plot analysis revealed that publication bias was unlikely, with no studies missing on the left side of the funnel plot (Figure 2). Indeed, the trim-and-fill algorithm suggested that, if anything, 78 ESs from 34 studies were missing at the *right* side of the funnel plot, suggesting a possible selection bias that excluded studies with *larger* ESs. After imputation of these missing ESs, an adjusted overall effect was estimated, which produced an average ES of *g*=0.40 (*P*<.001; 95% CI 0.33-0.46), somewhat larger than our initially estimated overall effect (Δ*g*=0.13). Nevertheless, it is important to note that the trim-and-fill analysis does not take the dependency in ESs into account. An Egger regression test, which better models dependencies among ESs, revealed that SE was a significant and positive predictor of ESs (*β*_1_=1.65, *P*<.001; 95% CI 1.07-2.23), which may indicate publication bias rather than selection bias.

**Table 2:** Moderators of the effectiveness of mobile technology–delivered interventions for youth: higher-quality studies only^a^.

Characteristics			*k^b^*	Effect sizes, n	B_0_ (intercept), *g* (95% CI)	B_1_ (slope), *g* (95% CI)	*F* test (df_1_, df_2_)		*P* value	
**Methodological characteristics**										
	Study quality		38	428	0.20 (0.11 to 0.29)^***^	–0.0005 (–0.03 to 0.03)	0.001 (1, 426)		.97	
	**Comparison group type**							3.25 (2, 425)		.04
		No intervention (RC^c^; eg, wait-list)	21	215	0.26 (0.18 to 0.35)^***^	N/A^d^				
		Inert (eg, placebo or information-only)	15	112	0.14 (0.05 to 0.23)^**^	–0.13 (–0.23 to –0.03)^*^				
		Clinical (eg, established intervention)	7	101	0.12 (–0.01 to 0.26)^e^	–0.14 (–0.29 to 0.01)^e^				
**Youth Characteristics**										
	Mean age (years)		36	407	0.19 (0.11 to 0.26)^***^	0.01 (–0.0002 to 0.03)^e^	3.75 (1, 405)		.05	
	**Risk level and type**							1.18 (3, 424)		.32
		General sample not selected for risk (RC)	17	175	0.13 (0.03 to 0.24)^**^	N/A				
		Nonmental health (ie, medical) risks	1	2	0.11 (–0.50 to 0.72)	–0.03 (–0.64 to 0.59)				
		Psychological or mental health at-risk sample	16	229	0.25 (0.15 to 0.36)^***^	0.12 (–0.03 to 0.27)				
		Psychological clinical sample (diagnosis)	4	22	0.31 (0.07 to 0.55)^*^	0.18 (–0.09 to 0.44)				
**Intervention characteristics**										
	**Prescribed frequency of use**							1.79 (4, 423)		.13
		As much as feasible; ≥4 days per week (RC)	22	306	0.23 (0.14 to 0.32)^***^	N/A				
		Some days, or more than once a week	5	27	0.22 (0.02 to 0.42)^*^	–0.01 (–0.22 to 0.21)				
		About once a week	3	36	0.40 (0.15 to 0.66)^***^	0.18 (–0.09 to 0.44)				
		One-time session	2	15	0.13 (–0.20 to 0.46)	–0.10 (–0.44 to 0.25)				
		Not stated, when needed, or at user discretion	6	44	0.03 (–0.14 to 0.20)	–0.20 (–0.39 to –0.01)^*^				

^a^This table presents moderation results for higher-quality studies (*k*=38) only in cases where the statistical significance of the moderation effect differs from the full-sample (*k*=80) results presented in Table 1. The right columns list the omnibus *F* test and *P* value for each moderation test. The middle columns list the intercept (B_0_), or mean effect size, and slope (B_1_), an estimated unstandardized regression coefficient, of the relevant Hedges *g* statistics, with CIs around each. Effects and slopes that differ significantly from 0 are denoted with asterisks in the intercept (B_0_) and slope (B_1_) columns, respectively. For categorical moderators, each intercept represents the mean effect of a category, whereas each slope represents the difference in the mean effect between the category and reference category. Depending on its sign, the slope of a continuous moderator represents an increase or decrease in the effect size with each unit increase in the variable.

^b^Number of studies with relevant ES data for a given row. In cases of multiple interventions or comparisons, some studies were counted in multiple rows; thus, these numbers sometimes exceeded 38. Owing to missing data, some counts fell short of 38. Further details on what was included in different categories of the included moderators are provided in the *Methods* section.

^c^RC: reference category.

^d^N/A: not applicable (as the slope represents a comparison with the reference category).

^e^*P*<.10.

*P<.05.

**P<.01.

***P<.001.

#### Timing of Outcome Assessment

There were no significant differences in ESs immediately after the intervention versus those at longer-term follow-up assessments (Table 1).

#### Outcome Type

The effectiveness of mTDIs varied as a function of the type of youth outcome that was targeted or assessed. The results in Table 1 indicate that there were statistically significant, positive effects of mTDIs on all of the coded outcome categories: general psychological distress or well-being, internalizing distress, noninternalizing mental health concerns, psychosocial strategies and skills, health-related outcomes, and *other* outcomes (see the *Methods* section). However, the *other* outcomes showed significantly lower ESs than the reference category, on average.

#### Comparison Group Type

Contrary to expectations, in the full sample, the comparison group type did not moderate ES, such that effects were not statistically different across studies using no-intervention (eg, wait-list) or inert (eg, placebo or information-only) comparison groups, as well as studies using clinical treatments as their comparison group (Table 1). However, among the higher-quality studies, the results were more in line with our hypotheses, in that studies using inert comparison groups produced lower ESs than studies using no-intervention control groups, and studies using a clinical comparison no longer showed statistically significant effects on youth outcomes (Table 2).

### Differences in Effects of mTDIs Based on Youth Characteristics

The results showed that the mean age of the youth participants did not moderate the impact of the mTDIs (Table 1). Among the higher-quality studies, there was an effect right at the *P*=.05 threshold, such that the older the mean age of participants, the stronger the effect (Table 2). There were no differences in the study ESs as a function of the youth gender breakdown in the sample. Missing data on race and ethnicity limited our ability to analyze this variable as a moderator.

Youth level and type of risk significantly moderated intervention effects in the full sample (Table 1), such that samples with nonmental health (ie, medical) risks showed larger effects of mTDIs than general, unselected youth samples. However, in the subsample of higher-quality studies, this moderating effect was not found; in fact, the medical risk category dropped to one study and was no longer significantly different from zero (Table 2).

### Differences in Effects of mTDIs Based on Intervention Characteristics

#### Primary Type of Technology

Moderation analysis did not detect statistically significant differences in the impact of mTDIs based on the primary type of technology: mTDIs were effective—and similar in their impact on youth outcomes—whether delivered on a smartphone or tablet, a presmartphone mobile device, or a mobile VR or handheld video game (Table 1).

#### Guiding Theoretical Framework

As hypothesized, both cognitive behavioral and mindfulness- or acceptance-based interventions (as well as interventions that blended these 2 orientations) had significant effects on youth outcomes. Interventions grounded in one or multiple other theoretical frameworks also yielded significant effects and did not appear to differ systematically in their effects from cognitive behavioral interventions. Those mTDIs that were atheoretical or did not specify a guiding theoretical framework did not significantly differ from zero in their impact on youth outcomes, and the CI around their intercept (mean effect) was quite wide, indicating considerable heterogeneity. Of note, these studies were rare (*k*=3), and all 3 studies were dropped from the analysis of higher-quality studies; however, the overall pattern of results remained the same.

#### Technological and Support Features

Exploratory analyses of the impact of intervention features and support failed to detect significant moderation of intervention effects based on the presence or absence of various technological features of the mTDI, including personalization, tailoring, social components, or gamification elements. Similarly, there were no differences in effects for mTDIs that integrated various support features, such as a training or orientation to the mTDI; some other in-person element; reminders to use the app; human or bot guidance, coaching, feedback in mTDI use; provision of supportive accountability; or supervised practice of skills taught by the mTDI. Although no significant differences were found between the absence and presence of any of these features and support types, it is notable that mTDIs both with and without each of these features had significant and positive mean effects (Table 1).

#### Dosage: Prescribed Frequency and Duration of Use

There was a significant moderation effect for the prescribed frequency of mTDI use (Table 1). All prescribed use frequencies, except for leaving use to user discretion (including unstated use prescriptions), had a statistically significant impact on youth outcomes. Those mTDIs that involved prescribed use about once per week or were a single session yielded higher ESs than the reference category, which involved prescriptions of more frequent mTDI use (ie, at least 4 days per week or as much as feasible). However, this effect was not retained in the sample of higher-quality studies: The 2 remaining studies that prescribed a one-time session no longer yielded ESs that differed from zero statistically, and the prescribed use frequency was no longer a significant moderator of ESs (Table 2).

Additional moderation analyses probing the number of prescribed weeks or sessions of mTDI use did not detect significant effects for the prescribed duration of the intervention, whether the number of intervention sessions or weeks (Table 1). Although we also intended to examine the moderating effect of completed dosage (frequency, weeks, and sessions), there were substantial missing data, precluding meaningful analysis of these moderators.

## Discussion

### Principal Findings and Comparisons With Prior Work

To our knowledge, this study represents the first review and meta-analysis of mTDIs for a wide variety of youth well-being outcomes, an area of research that has grown rapidly in the past decade. Rigorous searches of the published and unpublished literature in this area yielded 80 studies evaluating 83 mTDIs for youth. A 3-level meta-analysis revealed an overall Hedges *g* of 0.27 across all youth outcomes and follow-up assessments, indicating a small effect that is generally consistent with the observed impact of mTDIs in other meta-analyses [42,62,67-69]. This finding addresses a critical gap in the existing literature in that most previous meta-analyses have focused solely on the effects of mTDIs in adult populations [42,53,63], and the few studies focusing specifically on youth have aggregated across diverse types of mobile and nonmobile technologies [64,65] or limited their scope to a specific subset of youth disorders (eg, internalizing disorders [67]).

It is worth noting that our sample included many studies of mTDIs that were still relatively early in their development and were, therefore, primarily interested in evaluating the feasibility and acceptability of the technology, although they also included measures of more distal mental health outcomes that they ultimately aimed to influence. Therefore, our analyses may underestimate, to some extent, the impact that these mTDIs would have had in larger or longer efficacy trials more specifically designed to influence youth mental health outcomes. As this literature continues to mature, it will be important to focus the inclusion criteria more specifically on studies that measure the effects of mTDIs on more distal mental health outcomes as their primary focus.

Our publication bias analyses yielded conflicting findings that were difficult to interpret, given the lack of conventions for analyzing publication bias in 3-level models. However, it is worth noting that despite rigorous screening criteria for the methodology of included studies, our coding revealed significant variability in study quality, with less than half of the studies in our sample comprising effects that surpassed our defined standards for typical research practices [89]. Study quality appeared to significantly influence the observed ESs such that as the score for study quality increased, the ESs generally decreased. Only a few prior meta-analyses of TDIs have directly assessed the influence of study quality on ES and found no impact [57,201]. However, our findings are consistent with some previous findings linking study quality to observed ESs for other psychological interventions [202,203] and suggest that attention to rigorous experimental methods, such as the reporting of intent-to-treat analyses and the use of well-validated and reliable assessment tools, are essential to accurately identify the impact of mTDIs for youth.

#### Methodological Characteristics Affecting Outcomes

Given the substantial heterogeneity across ESs, both within and between studies, we explored several moderators as predictors of this variability. Interestingly, the ESs were similar in the immediate posttest assessments and longer-term follow-up assessments. This was true despite the fact that follow-up assessments occurred, on average, at 11.52 weeks, and ranged in length up to 43.14 weeks, after the active intervention period concluded. This finding is in contrast to the decrease in the effectiveness over time of some in-person mental health treatments [204-206] and suggests that the impact of mTDIs endures over time. It is possible that these enduring effects are because mTDIs are more easily integrated into youths’ lives, therefore leading to either greater generalizability of the intervention effects, more lasting engagement with the mTDI, or both.

Somewhat contrary to expectations, moderator analyses in the overall sample also revealed that mTDIs had a similar impact on youth outcomes regardless of whether they were compared with a no-intervention control, such as a wait-list, or a more active comparison group, such as an information-only condition or usual clinical care. This helps to rule out the effects of expectancies, demand characteristics, or nonspecific effects accounting for the benefits of mTDIs in youth. This finding contributes to the somewhat mixed literature on this topic, with some past reviews of TDIs with both youth and adult samples finding that, more generally, ESs tend to differ based on comparison type (eg, higher for wait-list vs more active comparisons) [45,52,58,69,70], whereas others indicate that TDIs tend to be similarly effective across various types of study designs (eg, the study by Farrer et al [85]). Indeed, even in this meta-analysis, some findings shifted when only higher-quality studies were analyzed, such that studies with information-only or placebo comparisons yielded lower ESs than studies with no-intervention control groups, and studies with clinical comparison groups no longer showed statistically significant effects. Moreover, it is worth noting that the specific nature of the comparison group varied quite widely across studies, even within a particular coded category. As such, future research should continue to explore the marginal benefits of mTDIs over other available interventions.

The positive impact of mTDIs was observed across the diverse youth outcome categories assessed by each study, with the largest ESs for psychosocial strategies and skills (eg, emotional self-awareness, self-efficacy, and coping) and internalizing symptoms such as depression and anxiety, followed by general psychological distress and well-being, health concerns and health-related behaviors, and other noninternalizing mental health concerns (eg, attention difficulties, aggression, or delinquency). The smallest ES was observed for *other* outcomes (eg, knowledge, peer relationship quality, and stereotype threat), which showed significantly smaller effects than the reference category of general psychological distress. However, it is difficult to interpret this finding, given that our *other* category contained a diverse set of outcomes, many of which were coded very infrequently. As such, the overall ES for this *other* category is not necessarily reflective of the lower impact of mTDIs on each of these less commonly coded outcome types, and future research should continue to explore the scope of the impact of mTDIs on diverse youth problems. Nevertheless, these findings regarding outcome types generally suggest that mTDIs can be effective in treating a wide array of problems across youth development, including diverse areas of psychopathology (ie, both internalizing and externalizing domains), in addition to a number of cognitive, behavioral, and social risk factors that are often associated with poor mental health. This is a significant contribution to the literature, which has previously focused on narrower sectors of outcomes when analyzing the effectiveness of TDIs and mTDIs for youth [51,67,68].

#### Youth Characteristics Affecting Outcomes

In the overall sample, there was no association between average youth age and the impact of mTDIs. However, when lower-quality studies were excluded from the analysis, the effects of youth age emerged more strongly, with ESs increasing as the mean participant age increased. This effect was right at the threshold for statistical significance (*P*=.05) and should, therefore, be interpreted with caution and replicated in future studies; however, this finding that suggests stronger effects of mTDIs for older youth is consistent with some past literature on TDIs more generally [51,54,55,65,72]. As many mental health interventions, including TDIs, were originally developed with adults in mind and only later adapted for youth at various stages of development, it is perhaps not surprising that mTDIs could have a more robust impact on older adolescents and young adults. For example, these youth may have greater internal motivation to engage with the intervention and be better able to interact with and adhere to the cognitive or behavioral skills taught by the mTDIs. However, given the complex ways in which developmental stages interact with risk for diverse mental health problems, as well as the effectiveness of mental health interventions, future research should continue to probe interactions between youth age and other dimensions of mTDIs (eg, level of human support, guiding theoretical framework, and availability of a social component) in predicting the impact of mTDIs.

Youth risk characteristics significantly moderated intervention effects in the full sample, with studies in which youth were selected for indicators of medical risk (eg, youth diagnosed with spina bifida or about to undergo surgery or another medical procedure) showing an average ES more than double that of studies with general, unselected samples of youth. Samples of youth with psychological risk (either clinically significant or subclinical risk) fell somewhere in the middle. However, in the analyses that excluded the lowest-quality studies, this moderating effect was no longer observed. The lack of differential findings for the impact of mTDIs on outcomes for youth with clinically significant versus subclinical risk, even when compared with general or unselected samples, is somewhat consistent with previous findings on TDIs, which tend to be quite mixed in terms of the impact of TDIs for youth with a variety of risk profiles [47,53,60,65]. Future research should continue to explore the presenting problems and risk indicators that are the best fit for referral to mTDIs versus more or less intensive interventions.

Other youth characteristics, in addition to age and risk factors, were not demonstrated to predict observed ESs. Youth race and ethnicity were reported inconsistently and according to widely varying conventions and, therefore, could not be tested as moderators of ESs. Furthermore, these identities and lived experiences are intertwined with structural inequalities and systems of discrimination and oppression that are more important for research to assess and relate to well-being outcomes. Consistent with several previous reviews [42,59], the breakdown of youth gender in the study sample did not predict ESs, although it is worth noting that studies infrequently made a note of nonbinary gender categories. These findings point to the need for a more careful and nuanced assessment of youth identities and lived experiences, including those connected to race, cultural identity, gender, sexual identity, and socioeconomic background, in studies testing the impact of mTDIs on youth.

#### Intervention Characteristics Affecting Outcomes

Smartphone- or tablet-based mTDIs were by far the most commonly reported primary technology in our sample of studies relative to presmartphone mobile devices or other (mobile VR and handheld video game) technologies. The type of technology did not appear to moderate ESs, with each of these types of mTDIs yielding average ESs that were statistically significant and of a similar size. Although our sample was limited in number, mobile VR technologies are promising avenues for further research, especially given the effectiveness of these technologies for conditions such as posttraumatic stress disorder, depression, and pediatric pain and anxiety during medical procedures [207,208]. Although no studies in our sample used wearable devices as the primary type of technology, a handful used a smartphone along with some sort of wearable biosensor such as a sleep monitor or a physical activity wristwatch [113,183,188,195]. As these technologies are likely to become more common over time, research should continue to explore their effectiveness as a primary or supplemental feature of mTDIs for youth well-being.

Consistent with the previous literature on TDIs and mTDIs for youth and adults [51,53,54,69], both cognitive behavioral and mindfulness- or acceptance-based interventions (as well as interventions that blended these 2 orientations) had significant effects on youth outcomes. In traditional in-person treatment settings, cognitive behavioral interventions, including third-wave cognitive behavioral treatments that include components of mindfulness and acceptance, have become increasingly popular as empirical support has grown for their effectiveness in treating a wide range of childhood disorders, including anxiety, depression, conduct or aggression problems, and attention difficulties [209]. However, a growing body of literature shows that many youths and families are not able to access these *gold standard* evidence-based treatments, whether because of lack of availability in their community or issues with accessing mental health care in general, such as cost and stigma [26,210,211]. Therefore, it is encouraging to see that the effectiveness of these evidence-based interventions can be translated into low-cost, mobile technology–delivered formats that can reach far larger numbers of youth, and perhaps in a way that is more generalizable to the naturalistic environments of their lives. Interventions in our sample that were grounded in one or multiple *other* theoretical frameworks, such as positive psychology or motivational interviewing, also yielded significant effects and did not appear to differ systematically in their effects from cognitive behavioral interventions. Although few studies have examined (m)TDIs using these theoretical approaches, these findings are consistent with previous research that has evaluated the impact of these specific theoretical orientations [202,212]. Our sample also included 3 mTDIs that did not specify a guiding theoretical framework [154,166,171], and collectively, they did not significantly differ from zero in their impact on youth outcomes. These findings should be interpreted with caution, given the small number of studies, wide CIs around their intercepts (mean effects), and the fact that these studies were excluded from the analysis of higher-quality studies. Future research should continue to explore the impact of mTDIs grounded in diverse theoretical frameworks. With the vast and rapidly growing number of available mTDIs purporting to support the well-being and mental health of youth, it is critical to ascertain the theoretical frameworks that may lend themselves best to developing active interventions with strong empirical support for their effectiveness in a mobile technology–delivered format.

As mTDIs have become increasingly popular, many have begun to incorporate additional technological features intended to better leverage the technology-based format to engage and sustain users’ attention. For example, some apps may personalize features of the intervention to the user’s personal preferences or tailor the intervention based on a user’s in-the-moment responses [75]. Others may include a social component, such as integration with social media platforms or a chat forum, or incorporate aspects of gamification, such as challenges or quests associated with points or badges [213]. Our moderator analyses showed no influence of these features on the ESs. However, it should be noted that many of these features are still relatively uncommon in mTDIs tested by research. For example, only 12% (10/83) of mTDIs in our meta-analysis mentioned a social component, and only 22% (18/83) described elements of personalization. Thus, the importance of these features may become more apparent as mTDIs targeting youth well-being begin to incorporate them more regularly and with greater proficiency. It is also likely that the most impactful mTDIs use an effective *combination* of these features to engage youth rather than simply incorporating one or another single design feature.

Given extensive theories [77,214,215] and some prior research on the benefits of outside guidance on engagement with mTDIs [53,57,216], we were somewhat surprised to see that the incorporation of support features, such as the provision of supportive accountability for technology use or supervised practice of the skills introduced by the mTDI, did not significantly moderate the ESs for mTDIs for youth. However, research in this area has been quite mixed, with several other studies finding little or no benefit from the inclusion of coaching or human support [42,54,55]. It is possible that mTDIs that do not rely on any component of human or bot support tend to be designed in a more comprehensive and self-contained way to offset this lack [42]. Moreover, the lack of significant moderation findings for these features in our meta-analysis does not necessarily indicate that these features are unimportant to the success of mTDIs in youth. Our meta-analysis captured an unusual sample of mTDI users, given that all effects were evaluated within the context of researcher-guided studies. As such, *all* participants were likely exposed to greater-than-usual accountability and support for their technology use as a function of taking part in a research study. This level of baseline accountability may have made it difficult to observe the added benefits of other forms of guidance or support. In addition, there are likely several kinds of informal human support—such as guidance or support from caregivers, teachers, or peers—that influence youth but were not typically assessed or reported in our sample of studies. Future research should continue to explore the kinds of support that are needed to maximize engagement with mTDIs for youth mental health and the ways in which these supports interact with factors such as youth age or risk characteristics (eg, younger or more clinically at-risk youth may require greater support).

Finally, moderation analyses provided tentative evidence that mTDIs can be effective regardless of the prescribed frequency or duration of use. In the overall sample, the number of prescribed weeks or sessions of use did not moderate the ESs. Moreover, mTDIs yielded significant ESs across all prescribed use frequencies, except for mTDIs that did not prescribe a use frequency or left use up to the user’s discretion. In the higher-quality sample, single-session interventions no longer yielded a significant ES; however, this finding should be interpreted with caution as only 2 single-session interventions remained in the higher-quality analytic sample.

These findings add to a growing body of mixed findings regarding the impact of prescribed and actual mTDI use on intervention outcomes [57,85,86]. Reviews of technology-based mental health interventions often highlight significant problems with treatment initiation and dropout [217-219], particularly for self-guided treatments that involve lower levels of structure and prescriptive guidance [220,221]. As such, it is critical for meta-analytic research to continue to explore trends in whether and how the prescribed dosage of mTDIs influences youth outcomes, with a particular focus on how different types of prescriptive guidance fit best with users’ specific needs. For example, youth with more severe clinical diagnoses may require a different dosage of mTDI than those engaging with a prevention-oriented mTDI designed to improve general well-being. Notably, there was wide variability in how studies reported on mTDI dosage and adherence. We chose to analyze the *prescribed* dosage and frequency of mTDI use, given that these statistics were most consistently reported. It would be ideal to analyze the actual *completed* use or uptake of mTDIs among participants as well; however, this was reported inconsistently among the studies. Several studies reported participant use only among study completers or dropped unengaged or low-engaged users from the analysis [121,127,152], whereas others used financial incentives for protocol compliance [151], potentially introducing bias into the use statistics recorded in research studies that have additional levels of accountability built into the protocol.

### Limitations and Future Directions

The results of this meta-analysis should be interpreted with several limitations in mind. First, the quality of any meta-analysis is limited by the quality of the available primary studies. Only 60% of our coded ESs, which came from 48% (38/80) of the included studies, met our high-quality standards. We attempted to address this issue by running all analyses on both the full sample of studies and a subsample of higher-quality studies. Nevertheless, as a substantial number of studies and ESs of lower quality were dropped from the higher-quality subsample analysis, the statistical power declined, and some moderator categories could not be examined. Future research in this area should attend to existing procedures for designing and reporting on high-quality clinical intervention research. For example, there is a need for more studies that use larger sample sizes and retain larger percentages of their participants (regardless of their mTDI engagement), include more reliable outcome measurements, and use intent-to-treat analyses, as well as studies conducted by authors who were not involved with app development and are, therefore, able to provide a more unbiased assessment of the mTDI’s impact.

Relatedly, our coding scheme yielded incomplete data for many of our hypothesized moderators, given the variability in reports on characteristics of the tested mTDI (eg, human support features, duration and frequency of use), as well as youth characteristics (eg, race and ethnicity, gender, and risk characteristics). Therefore, additional studies that carefully document these kinds of data are needed to more thoroughly test the various moderators of the overall effects of mTDIs.

Given the lack of standard approaches for assessing publication bias within a 3-level meta-analysis, we applied 2 different techniques that produced conflicting findings. The trim-and-fill analysis pointed toward a potential underestimation of the true overall effect, whereas the Egger test pointed toward a potential overestimation of the true overall effect (and thus publication bias). The Egger test—in which ES dependencies are modeled—is likely more valid than the trim-and-fill results for our multilevel study. Nevertheless, the results of both techniques should be interpreted with caution, as neither was developed for a 3-level meta-analysis, and both rely on an assumption of homogeneity in ESs, which is often not met in meta-analytic studies, including this study.

As noted previously, another limitation in interpreting the present findings is that youth mTDI use likely differs within versus outside of a research study. At the very least, research participants tend to be much more informed about intervention goals and receive more structured support in the process of using an mTDI than users outside the research context. In more naturalistic settings, such as clinical practice or completely self-guided use, mTDIs are likely to be used much more flexibly and may be adapted to the needs and circumstances of individual youths.

When considering the implications of the present findings for the use of mTDIs in clinical and other naturalistic settings, it is also essential to consider issues such as accessibility and cultural sensitivity of mTDIs, which were not examined in this meta-analysis. Some types of mTDI technologies, such as VR headsets, may be prohibitively expensive and less readily accessible to some youth and families, particularly those living or seeking treatment in low-resource settings. Moreover, wide variability in families’ digital literacy and cultural norms around using technology to improve well-being is likely to play a key role in the effectiveness of these types of interventions. The studies in this meta-analysis were largely limited to Western cultural contexts, with very little research emerging from certain parts of the world (eg, our search did not yield any research from the African continent). In addition, reporting on youth characteristics such as socioeconomic status, race, and ethnicity was quite limited and followed widely varying conventions. Taken together, these issues limited our ability to test questions related to the cultural responsivity or tailoring of particular mTDIs based on youth cultural backgrounds—important questions that future research will need to explore to fulfill the promise of mTDIs for youth living in communities traditionally underserved by available in-person prevention and intervention programs.

In addition, participant ratings of social validity (ie, acceptability of mTDIs and user satisfaction), among the 25% (20/80) of studies for which these data were available, averaged under 60%, indicating that these technologies likely have room for improvement in user interface and user experience design. Further research should more diligently assess for different aspects of social validity, including qualitative feedback, and relate these elements to outcomes and potential moderators (eg, age of users, in-app features, and clinical severity of users), with the ultimate goal of improving the engagement and uptake, and thus impact, of mTDIs for youth.

Finally, future research should continue to explore the ideal setting and level of support for various mTDIs. The optimal approaches to integrating mTDIs with other mental health tools, as well as face-to-face interaction with mental health providers, remain largely an open question at this time. Although in some cases, mTDIs may serve as low-cost and accessible substitutions or adjunctive supports for face-to-face intervention programs in areas with limited access to health professionals, mTDIs may also be valuable as a way of socializing some youth to psychosocial interventions, with the goal of eventually connecting families to more traditional face-to-face services. In other cases, mTDIs may be most useful when accompanied by the support of a clinician or paraprofessional, such as a teacher, mentor, or academic advisor who guides the youth through the technology-based intervention [118,120,158].

### Study Strengths and Conclusions

This is the first comprehensive 3-level meta-analysis to evaluate the effects of mTDIs on diverse aspects of well-being in youth. We built on prior work by taking an inclusive but rigorous approach to testing the impact of interventions using various types of mobile technologies on diverse outcomes across childhood, adolescence, and young adulthood. Using a 3-level approach to meta-analysis, we were able to synthesize all relevant ESs while accounting for both within- and between-study heterogeneity in ESs and maximizing the statistical power of the analyses. Moreover, we coded a comprehensive set of more than 3 dozen potential moderators of study effects and found sufficient information to analyze the moderating role of >20 of these variables, including technological and support features (eg, human support and availability of in-app reminders) that are hypothesized to be critical to the success of these interventions but have rarely been tested as predictors of effects in previous meta-analyses.

Our synthesis of primary research confirms the significant benefits of mTDIs across a variety of psychosocial outcomes, comparison types (ie, no intervention, inert, and clinical), and time points (both immediate postintervention and longer-term follow-up effects). Although additional high-quality research on which kinds of mTDIs are most effective and under what conditions is clearly needed, we conclude that mTDIs have the potential to improve multiple aspects of youth well-being, and may confer significant, durable benefits in a broad array of domains, particularly for youth who are not otherwise getting their mental health needs met.

## Appendix

Multimedia Appendix 1 Additional search strategy details.

Multimedia Appendix 2 Selected characteristics of 80 studies evaluating 83 mobile technology-delivered interventions for youth.

## Abbreviations

ES: effect size
mTDI: mobile technology–delivered intervention
PRISMA: Preferred Reporting Items for Systematic Reviews and Meta-Analyses
TDI: technology-delivered intervention
VR: virtual reality
