# Supplementary material for: The Impact of Mobile Technology-Delivered Interventions on Youth Well-being: Systematic Review and 3-Level Meta-analysis
Source: JMIR Ment Health. 2022 Jul 29;9(7):e34254. doi: 10.2196/34254 (PMC9377434; doi:10.2196/34254)
Supplement: Multimedia Appendix 1 [file mental_v9i7e34254_app1.pdf]

Conley, C.S., Raposa, E.B., Bartolotta, K., Broner, S.E., Hareli, M., Forbes, N., Christensen, K.M., & Assink, M. (2022). The impact of mobile technology-delivered interventions on youth well-being: Systematic review and 3-level meta-analysis. *JMIR Mental Health, 9(7):e34254 URL: https://mental.jmir.org/2022/7/e34254/ doi: 10.2196/34254*

Multimedia Appendix 1. Additional Search Strategy Details

Comprehensive List of Journals (and Years) Searched (Full Table of Contents)

- Annual Review of CyberTherapy and Telemedicine; 2010-2020
- Clinical Psychological Science (CPS); 2013-2020
- Computers in Health Care; 2010-2015
- Computers in Human Behavior; 2010-2020
- Cyberpsychology and Behavior (CB); 2010-2020
- Internet Interventions (II); 2014-2020
- International Journal of Medical Informatics (IJMI); 2010-2020
- Journal of Abnormal Psych (JAP); 2010-2020
- Journal of Clinical Child and Adolescent Psych (JCCAP); 2010-2020
- Journal of Consulting & Clinical Psych (JCCP); 2010-2020
- Journal of Counseling Psych (JCP); 2010-2020
- Journal of Cybertherapy and Rehabilitation (JCR); 2008-2010
- Journal of Medical Internet Research (JMIR)-Mental Health; 2014-2020
- Journal of Medical Internet Research (JMIR)-Pediatrics and Parenting; 2018-2020
- Journal of Technology in Human Services; 2010-2020
- Studies in Health Technology and Informatics; 2010-2020

Comprehensive List of Conference Proceedings (and Years) Searched

- American Psychological Association Division 54 - Society for Pediatric Psychology 2015-2020
- Anxiety and Depression Association of America (ADAA) 2015-2020
- Association for Behavioral and Cognitive Therapies (ABCT) 2015-2020
- Association for Contextual Behavioral Science (ACBS) 2015-2020
- Association for Psychological Science (APS) 2015-2020
- The International Society for Research on Internet Interventions (ISRII) 2013-2019 (no conference 2020)
- Society of Behavioral Medicine (SBM) 2015-2020

Comprehensive list of professional organizations contacted (e.g., through email lists, social media sites) to ask for work that could be included in the meta-analysis.

- Association for Behavioral and Cognitive Therapies (ABCT)
- American Psychological Association (APA) Technology, Mind, and Society
- APA Division 53 - Society of Clinical Child and Adolescent Psychology (SCCAP)
- APA Division 54 - Society for Pediatric Psychology
- Society for the Study of Emerging Adulthood (SSEA)
- The International Society for Research on Internet Interventions (ISRII)
- Society of Behavioral Medicine (SBM)

Conley, C.S., Raposa, E.B., Bartolotta, K., Broner, S.E., Hareli, M., Forbes, N., Christensen, K.M., & Assink, M. (2022). The impact of mobile technology-delivered interventions on youth well-being: Systematic review and 3-level meta-analysis. *JMIR Mental Health, 9(7):e34254* *URL:* https://mental.jmir.org/2022/7/e34254/ *doi:* 10.2196/34254

List of Databases Searched (Through March 2021)

- PsycInfo
- ERIC
- ProQuest Digital Dissertations
- MEDLINE (Web of Science)
- PubMed

Search Terms for Electronic Databases

Boolean searches were conducted as follows, to capture reports that included at least one term from each category (population, technology, intervention, mental health, and study design/type):

(“teen*” or “young people” or “youth*” or …) AND (“MHapp” or “Smartphone tech*” or “e-health” or …) AND (“RCT*” or “PRISMA*” or “allocat*” or ...) AND (“cognitive training” or “mood monitor*” or “cognitive behavioral” or ...) AND (“mental health*” or “depress*” or “Panic disorder” or ...)

Population	Technology	Intervention	Mental Health
• teen*<br>• young people<br>• youth*<br>• 0 to 18 years<br>• adolescen*<br>• child*<br>• parent*<br>• caregiver*<br>• emerging adult<br>• undergraduate<br>• college student<br>• university student<br>• higher education student<br>• community college<br>• vocational school<br>• transition to adulthood<br><br>**Study Design/Type**<br><br>• RCT<br>• PRISMA<br>• allocat*<br>• random*<br>• trial*<br>• quasi*<br>• systematic literature review<br>• meta-analy*<br>• systematic review<br>• intervention*<br>• controlled trial<br>• comparison group<br>• control group<br>• clinical trial	• MHapp<br>• Smartphone tech*<br>• e-health<br>• mhealth*<br>• smartphone app*<br>• mobile phone app*<br>• cell phone app*<br>• mobile app*<br>• cellular phone app*<br>• mobile*<br>• mobile health<br>• tablet-based<br>• smartphone mental health application<br>• electronic device<br>• electronic application<br>• eCBT<br>• iCBT<br>• cCBT<br>• computer-delivered CBT<br>• Internet-delivered CBT<br>• handheld PC<br>• personal digital assistant<br>• palm pilot<br>• blackberry<br>• Gizmo<br>• Gabb<br>• MyPal<br>• smart watch<br>• smartwatch<br>• wearable device<br>• iphone<br>• android<br>• ipad<br>• smartphone<br>• mobile phone<br>• cell phone<br>• mobile application<br>• phone application	• cognitive training<br>• mood monitor*<br>• cognitive behavioral<br>• App-supported smartphone intervention*<br>• psychological treatment<br>• computerized treatment<br>• exposure<br>• treatment as usual<br>• pre-treatment assessment<br>• post-treatment assessment<br>• mindfulness*<br>• relaxation<br>• meditation<br>• behavior modification<br>• behavioral activation<br>• cognitive behavior*<br>• stress management<br>• parent training<br>• coping<br>• skill-building<br>• wellness-promoting<br>• intervention<br>• treatment<br>• effective*<br>• efficac*	• mental health*<br>• depress*<br>• Panic disorder<br>• Obsessive-compulsive*<br>• anxiety*<br>• distress<br>• positive affect<br>• generalized anxiety*<br>• social anxiety*<br>• post traumatic stress*<br>• negative affect<br>• m-health<br>• agoraphobia<br>• phobia<br>• panic<br>• post-traumatic stress<br>• affective*<br>• bipolar<br>• psycho*<br>• schizophreni*<br>• self-harm<br>• self-injur*<br>• stress*<br>• mood*<br>• body image<br>• eating disorder<br>• substance use<br>• PTSD<br>• sleep problems<br>• suicidal*<br>• alcohol use<br>• insomnia<br>• emotional disorders<br>• psychiatric*<br>• somatic<br>• behavioral disorder<br>• conduct disorder<br>• well-being<br>• Wellbeing
