# Supplementary material for: The Impact of Mobile Technology-Delivered Interventions on Youth Well-being: Systematic Review and 3-Level Meta-analysis
Source: JMIR Ment Health. 2022 Jul 29;9(7):e34254. doi: 10.2196/34254 (PMC9377434; doi:10.2196/34254)
Supplement: Multimedia Appendix 2 [file mental_v9i7e34254_app2.pdf]

Multimedia Appendix 2. Selected Characteristics of 80 Studies Evaluating 83 Mobile Technology-Delivered Interventions for Youth

Study	Age Range *M(SD)*	*N*	Sample Characteristics	Intervention Description	Intervention Features	Prescribed [Completed][a] Dosage (Frequency, Duration) *Average unless otherwise noted*	Outcome Types	Comparison Group(s)
Anastasiadou et al., 2020 [112]	12 and above 18.06 (6.04)	106	Spanish youth diagnosed with an eating or feeding disorder	TCApp, a cognitive-behavioral app, intended to connect patients with therapists in between appointments and increase patient engagement with CBT	GAM, ORT, F2F, REM, COA, SAC	Daily for 12 weeks [7.11 weeks]	GWB, INT, HTH	Clinical [In-person CBT]
Antle et al., 2018 [113]	5-11 ---	22	Nepalese girls with a history of trauma, living in poverty	Mind Full app using a neurofeedback brain-computer interface aimed at improving self-regulation of anxiety and attention	TLR, GAM, ORT, COA, SAC	24, 15-minute sessions conducted 3-4 times per week over 6 weeks [25.70 total sessions, for 8.43 minutes, conducted over 6 weeks]	SKL, OTH, APP	Clinical [In-person instruction in breathing practices and yoga]
Arps et al., 2018 [114]	16-21 18.09 (1.70)	136	New Zealand older adolescents and young adults	Gratitude text messaging, a positive psychology intervention prompting participants to respond to gratitude questions	REM	Daily text messages for 28 days [Throughout the 4 weeks, the majority of the gratitude questions were answered]	GWB, INT, SKL	Inert [Reflective text messages]
Baskerville et al., 2018 [115]	19-29 ---	1,599	Canadian young adult cigarette smokers intending to quit within the next 30 days	Crush the Crave (CTC), a behavior change app intended to reduce smoking prevalence among young adult smokers	PER, TLR, SOC, GAM, REM	Use as needed for 6 months [At 6 months, 359 participants had downloaded the app and 351 self-reported that they used it frequently]	HTH, APP	Inert [Self-help booklet]
Beidel et al., 2021 [116]	7-12 9.55 (1.84)	42	United States children with a primary diagnosis of Separation Anxiety Disorder	Pegasys-VR™, a serious game, web-based app for use on an iPad, intended to decrease social anxiety and improve social skills	TLR, GAM, COA, SKP	24 sessions conducted twice weekly - 12, 3-hour sessions and 12, 1-hour sessions for a total of 48 hours of treatment [Not reported]	GWB, INT, OTH	Clinical [In-person Social Effectiveness Therapy for Children (SET-C)]
Bohleber et al., 2016 [117]	--- 16.90 (1.73)	941	Swiss employed and unemployed adolescents	Companion App, utilizing a peer mentoring system to promote well-being and reduce stress	PER, SOC, ORT, REM, COA, SAC	43.45 weeks, prescribed as much as needed [Not reported]	GWB, OTH, APP	No intervention

Study	Age Range *M(SD)*	*N*	Sample Characteristics	Intervention Description	Intervention Features	Prescribed [Completed][a] Dosage (Frequency, Duration) *Average unless otherwise noted*	Outcome Types	Comparison Group(s)
Borjalilu et al., 2019 [118]	--- 24.38 (3.01)	68	Iranian university students in a medical sciences program	**Intervention group 1:** Aramagar app, aimed to reduce stress through a mindfulness-based intervention **Intervention group 2:** Aramagar app (same as above) plus in-person therapy	**Intervention group 1:** TLR, F2F, REM **Intervention group 2:** TLR, REM	2.86 weeks, prescribed for daily use [Not reported]	GWB, INT	Clinical [In-person mindfulness workshops]
Breitenstein et al., 2016 [119]	2-5 2.93 (0.87)	1,238	United States, predominantly African American and Hispanic children from low-income urban communities receiving medical care at a pediatric primary care site	ezParent program, a tablet-based parent training program intended to promote parenting competence and improve child behavioral problems	GAM, ORT, REM	6, 1-hour modules conducted once every 2 weeks (biweekly) over 12 weeks [85.40% (34/40) modules completed over 12 weeks]	OMH, OTH, APP	**Group 1:** Clinical [In-person parenting intervention] **Group 2:** [Inert: Website with health promotion information for parents]
Broglia et al., 2019 [120]	19-35 21.95 (3.68)	38	United Kingdom help-seeking university students with anxiety or depression	Pacifica, a cognitive-behavioral app intended to address anxiety and low mood	SOC, F2F, REM, COA, SAC, SKP	App use as much as feasible between counseling sessions for 3 months [Not reported]	GWB, INT, SKL, HTH, OTH	Clinical [In-person counseling]
Bruehlman-Senecal et al., 2020 [121]	18.10-19.77 18.68 (0.35)	221	United States incoming first-year university students, about half of which with high levels of loneliness	Nod, a positive psychology, mindfulness, and cognitive-behavioral app intending to address loneliness	REM	App use as much as feasible for 4 weeks [Excluding 4 non-users, participants accessed 36.69 pages of content, completed 0.89 challenges, and clicked through 1.13 reflections]	INT, HTH, OTH, APP	No intervention [Waitlist]
Choi et al., 2020 [122]	7-12 10.88 (1.69)	36	Korean children with spina bifida	Glowing Stars[TM], a cognitive-behavioral app intended to promote self-management among children with spina bifida	F2F, COA, SAC	Daily use for 4 weeks [Not reported]	SKL, HTH, OTH	**Group 1:** Clinical [In-person IEP] **Group 2:** No intervention

Study	Age Range *M(SD)*	*N*	Sample Characteristics	Intervention Description	Intervention Features	Prescribed [Completed][a] Dosage (Frequency, Duration) *Average unless otherwise noted*	Outcome Types	Comparison Group(s)
Chow et al., 2017 [123]	7-13 9.15 (1.96)	40	Canadian children undergoing outpatient elective surgery at a local children's hospital	Story-Telling Medicine (STM), a transtheoretical app intended to reduce children's preoperative anxiety	--	Use as desired for 7-14 days as well as the 20-minute intervention on the day of surgery [Over half of participants accessed the app at home and most used the app on the day of the surgery]	INT	Clinical [In-person preoperative preparation by a child-life specialist]
Clarke et al., 2016 [124]	--- 19.09 (2.54)	36	Australian young adults from the University of Western Australia School of Psychology with high levels of insomnia	Attentional bias modification task, utilizing cognitive-behavioral techniques through a smartphone	--	5 consecutive nights prior to bed [Not reported]	INT, HTH	No intervention
Conley et al., (in prep) [125]	18-24 19.16 (1.34)	89	United States college students with elevated depression	Headspace, a mindfulness-based app, plus small-group peer supportive accountability	SOC, GAM, ORT, REM, COA, SAC	8.5 weeks, daily sessions [7.9 weeks, 28 sessions, (~half the days), 8.3 minutes per session]	GWB, INT, SKL, HTH, OTH, APP	No intervention [Waitlist]
Cumino et al., 2017 [126]	4-8 5.34 (1.08)	63	Brazilian children scheduled to undergo minor-to-moderate elective surgical procedures with general anesthesia	Six game-based smartphone app options used as cognitive distractions to manage preoperative anxiety	F2F	1, 30-minute single-session intervention [Assume completed as prescribed]	INT	**Group 1:** Inert [Verbal information about anesthetic procedure] **Group 2:** Inert [Verbal and written information about anesthetic procedure]
de Niet et al., 2012 [127]	7-12 9.90 (1.30)	141	Dutch children classified as overweight and obese	Short Message Service [SMS] Maintenance Treatment (SMSMT) administered via text messages on mobile phones, aimed to promote behavioral skills	TLR, ORT, REM, COA, SAC, SKP	Texting once per week for 9 months [0.76 texts/week in the first 3 months, then dropped to 0.51 texts/week for 70% of sample (while the other 30% of sample dropped out)]	HTH	Clinical [In-person family-based behavioral group intervention]

Study	Age Range *M(SD)*	*N*	Sample Characteristics	Intervention Description	Intervention Features	Prescribed [Completed][a] Dosage (Frequency, Duration) *Average unless otherwise notec*	Outcome Types	Comparison Group(s)
Deluca et al., 2021 [128]	14-17 15.17 (1.04)	883	United Kingdom adolescents with low-risk drinking behaviors	Electronic Brief Intervention (EBI), a smartphone app based on the FRAMES model (motivational interviewing, cognitive and behavioral components) for brief alcohol intervention	TLR, GAM, ORT, F2F, REM	Encouraged to use the app as much as needed [Only 103 (35%) participants in the eBI group engaged with the intervention]	HTH	**Group 1:** Clinical [1 session of in-person Personalized Feedback and Brief Advice] **Group 2:** No Intervention
Earle et al., 2018 [129]	18 and above ---	183	United States sample of racially and ethnically diverse first-year college students	CampusGANDR (Gamified Alcohol Norm Discovery & Readjustment), a cognitive-behavioral app aimed at reducing normative beliefs about alcohol and alcohol use	PER, TLR, SOC, GAM	Once per week over a 6-week period [Not reported]	HTH	Inert [App with non-alcohol related game content]
Egilsson et al., 2021 [130]	15-16 15.61 (0.26)	41	Icelandic high school students	SidekickHealth, a social health game app, designed to promote mental health, physical activity, and healthy food and drink	TLR, SOC, GAM, ORT	Complete health exercises as much as feasible during each week of 6-week intervention [129.88 sessions completed over the course of the 6 weeks]	INT, SKL, HTH, APP	No intervention [Waitlist]
Elicherla et al., 2019 [131]	7-11 ---	50	Indian children without any prior experience with the dental environment or treatment process	Little Lovely Dentist, a behavior guidance app intended to manage dental anxiety and fear	--	One-time use right before dental procedure [Assume completed as prescribed]	GWB, INT	Inert [Tell-show-do (TSD) in-person dental technique]
Fish & Saul, 2019 [132]	18-48 21 (---)	91	United States predominantly female university students	Headspace, a mindfulness-based app	--	10, 10-minute sessions, conducted over the course of 2 weeks [Participants submitted screenshots to verify they completed the 10 sessions, averaging 7 minutes per session]	INT	No intervention

Study	Age Range *M(SD)*	*N*	Sample Characteristics	Intervention Description	Intervention Features	Prescribed [Completed][a] Dosage (Frequency, Duration) *Average unless otherwise noted*	Outcome Types	Comparison Group(s)
Fitzpatrick et al., 2017 [133]	18-28 22.20 (2.33)	70	United States predominantly Caucasian university students with self-identified symptoms of depression and anxiety	Woebot, an automated conversation agent accessed via an instant messenger app (on a mobile device or computer), delivering CBT via brief, daily conversations and mood tracking	TLR, ORT, REM, COA, SAC	Up to 20 daily check-ins, lasting from 90 seconds to 10 minutes, over 2 weeks [12.14 check-ins over 2 weeks]	GWB, INT, APP	Inert [eBook with NIMH resources on college student depressive symptoms]
Flett et al., 2020 [134]	17-20 17.87 (0.47)	250	New Zealand first-year college students	Headspace, a mindfulness-based app	GAM, ORT, REM	To be used as needed for 3 months [Meditated for 79.32 minutes total, completed 7.91 sessions, practiced over 23.07 days]	GWB, SKL, OTH, APP	No intervention [Waitlist]
Gajecki et al., 2014 [135]	--- 24.72 (4.81)	1,932	Swedish university students with self-reported hazardous levels of alcohol consumption	**Intervention group 1:** PartyPlanner, a cognitive-behavioral mobile app designed to modify drinking intentions via simulations of drinking events followed by real-time eBAC visualizations **Intervention group 2:** Promillekoll, a cognitive-behavioral app aimed to reduce risky alcohol consumption, based on registering alcohol consumption in real time with instant visual eBAC	TLR	To be used as needed for 6 weeks [Self-report only: **Intervention group 1:** 41.40% said they used the app during the study period **Intervention group 2:** 74.10% said they used the app during the study period]	HTH, APP	No intervention
Gajecki et al., 2017 [136]	--- 25.70 (6.46)	237	Swedish university students with excessive alcohol consumption	TeleCoach™, a transtheoretical (motivational and behavioral) app involving monitoring of alcohol consumption and relapse-prevention skills training	--	To be used as needed for 12 weeks [Not reported]	HTH	No intervention [Waitlist]
Gibson, 2015 [137]	18 and above ---	48	United States undergraduate students with likely ADHD	Focus Check, a self-monitoring of attention app intended to improve attention regulation	ORT, REM	1 hour daily app use for 2 weeks in addition to experience sampling surveys 3 times daily for the first 5 days after the introductory session [42 responses over the course of 2 weeks]	OMH, SKL, APP	No intervention [Waitlist]

Study	Age Range *M(SD)*	*N*	Sample Characteristics	Intervention Description	Intervention Features	Prescribed [Completed][a] Dosage (Frequency, Duration) *Average unless otherwise noted*	Outcome Types	Comparison Group(s)
Gipson et al., 2019 [138]	18-26 20.61 (2.05)	120	United States college students with no previous medical diagnosis of a sleep disorder	Text messaging intervention guided by social-cognitive and behavior change theories, intended to promote sleep hygiene and improve sleep	REM, COA	12 text messages sent twice per week over the course of 6 weeks [111 (92.50%) participants completed the 6-week intervention]	HTH, OTH	Inert [Attention control group receiving biweekly text messages on healthy behaviors]
Glissmann, 2018 [139]	18-48 21.18 (4.87)	109	United States, predominantly Caucasian university students with elevated self-reported stress scores	Calm, a mindfulness meditation smartphone app	ORT	10-minute sessions conducted daily over 8 weeks [38 minutes of meditation per week over 8-week study]	HTH, APP	No intervention [Waitlist]
Gonzales et al., 2014; 2016; 2019 [140-142]	14-25 20.40 (3.50)	80	United States ethnically and racially diverse group of adolescents and young adults who recently completed treatment for substance abuse	ESQYIR (Educating & Supporting inQuisitive Youth In Recovery), a smartphone text messaging intervention that incorporates the principles from social-cognitive theory and CBT	TLR, REM	Daily for 12 weeks [Not reported]	HTH	Clinical [12-16 week in-person cognitive-behavioral relapse prevention program]
Grassi et al., 2007; 2009 [143-144]	20-25 23.27 (1.38)	60	Italian college commuter students	Video narrative guiding participants through a meditation experience on a mobile pre-smartphone	--	4, 10-minute sessions over 2 days [Not reported]	GWB, INT, SKL	No intervention
Grassi et al., 2011 [145]	20-23 20.86 (1.27)	60	Italian female university students	Stress Inoculation Training (SIT), a cognitive-behavioral based protocol, delivered via Universal Mobile Telecommunications Service (UMTS), intended to induce emotional changes and reduce exam anxiety	--	1 evening session every day for 6 days [Not reported]	GWB, INT	No intervention

Study	Age Range *M(SD)*	*N*	Sample Characteristics	Intervention Description	Intervention Features	Prescribed [Completed][a] Dosage (Frequency, Duration) *Average unless otherwise noted*	Outcome Types	Comparison Group(s)
Greer et al., 2019 [146]	18-29 25 (2.90)	51	United States predominantly Caucasian young adults with a cancer diagnosis and completion of cancer treatment within 5 years of starting the study	Vivibot, a chatbot delivered over Facebook messenger, using cognitive-behavioral intervention to increase positive emotion	TLR, COA, SAC	28 daily sessions conducted over 4 weeks [73.80 minutes across 12.10 sessions during 4 weeks of study]	GWB, INT, APP	Inert [Daily emotion ratings on Facebook messenger app]
Haug et al., 2013 [147]	--- 18.20 (2.30)	755	Swiss adolescents who smoked, just under 50% of whom had an immigrant background	SMS-Coach, a Short Message Service (or text message) intervention primarily based on the Health Action Process Approach (HAPA) to promote smoking cessation	PER, TLR, ORT, COA, SAC	Text messages sent at least 3 times per week over the course of 3 months [Replied to 6.50 out of 11 weekly SMS text message assessments, conducted over the course of 3 months]	SKL, HTH, APP	No intervention
Haug...Gmel, 2013 [148]	15 and above 17.95 (2.25)	477	Swiss vocational students, over 50% of whom had an immigrant background	Alk-Check, a social norms web- and text messaging-based intervention intended to reduce problem drinking	PER, TLR	Up to 3 text messages per week over the course of 12 weeks [Self-report only: Of participants with valid follow-up data, 94.40% indicated regularly receiving text messages. Of these, 49.80% indicated thoroughly reading, 44.60% reported taking a quick look at, and 5.60% did not read the messages]	HTH, APP	No intervention

Study	Age Range *M(SD)*	*N*	Sample Characteristics	Intervention Description	Intervention Features	Prescribed [Completed][a] Dosage (Frequency, Duration) *Average unless otherwise noted*	Outcome Types	Comparison Group(s)
Haug et al., 2017 [149]	--- 16.80 (1.60)	1,041	Swiss vocational and upper secondary students, just under 50% of whom had an immigrant background	MobileCoach Alcohol, a social norms web- and text messaging-based intervention intended to reduce problem drinking	TLR, SOC, ORT	119 total different text messages sent across participants 1-3 times weekly over the course of 3 months [Self-report only: Of participants with valid follow-up data, 94.10% indicated regularly receiving text messages. Of these, 65.60% indicated thoroughly reading, 32.60% reported taking a quick look at, and 1.70% did not read the messages]	HTH, APP	No intervention
Hides et al., 2019 [150]	16-25 19.90 (2.50)	169	Australian adolescents and young adults with elevated emotional distress within the past month	Music eScape, a smartphone app incorporating principles from the information-motivation-behavioral skills health behavior model	PER, TLR, REM	Use as needed for 1 month [Not reported]	GWB, SKL, APP	No intervention [Waitlist]
Hilt & Swords, (in prep) [151]	12-15.70 13.72 (0.89)	52	United States adolescents with elevated trait rumination	CARE mindfulness app targeting rumination	TLR, ORT, REM	3 weeks, 2 sessions per day of 1-12 minutes [1.60 sessions per day, 1.40 minutes per session]	INT, SKL, APP	Clinical [App with ecological momentary assessment (EMA) self-monitoring]
Huberty et al., 2019 [152]	18 and above 21.18 (4.87)	109	United States full-time undergraduate students with elevated stress	Calm, a mindfulness meditation app	ORT, REM	10-minute sessions conducted daily over the course of 8 weeks [37.90 minutes of meditation per week over the course of 8 weeks]	GWB, SKL, APP	No intervention [Waitlist]

Study	Age Range *M(SD)*	*N*	Sample Characteristics	Intervention Description	Intervention Features	Prescribed [Completed][a] Dosage (Frequency, Duration) *Average unless otherwise noted*	Outcome Types	Comparison Group(s)
Johnson et al., 2014 [153]	3-8 10.30 (5.10)	32	United States predominantly White children with a diagnosis of autism, Asperger's, or Pervasive Developmental Disorder-Not Otherwise Specified (PDD-NOS) with an existing order for medical imaging	Going to Imaging, a social script iPad app designed to improve behavior, compliance, and social functioning of children with autism during imaging procedures	--	1 session for 5 minutes [Assume completed as prescribed]	INT, OMH, OTH	Inert [Answering parent/child questions about imaging procedure]
Kajitani et al., 2020 [154]	--- 21.75 (2.60)	68	Japanese college and graduate students	Mental App, a smartphone app designed to assist college students with self-monitoring and self-screening for mental health disorders	TLR	To be used as needed for 2 weeks [Among those who chose to use the app, screen time of 9.03 minutes, for 5.66 days]	GWB, INT, OTH, APP	No intervention
Kauer et al., 2012; Reid et al., 2011; 2013 [155-157]	14-24 18.05 (3.20)	118	Australian adolescents with psychological distress or mental health concerns	The Mobiletype Program (Mobile Tracking of Young People's Experiences), a cognitive-behavioral self-monitoring app that assesses 8 areas of functioning	ORT, REM	2, 1-3-minute entries per day for 2-4 weeks [3.30 entries per day for 17.70 days]	GWB, INT, SKL	Inert [Attention-comparison group monitoring daily activities using abbreviated mobiletype app]
Kennard et al., 2018 [158]	12-18 15.10 (1.50)	66	United States adolescents who presented to a psychiatric inpatient unit with recent suicidal ideation or attempt	ASAP (As Safe As Possible), inpatient intervention for suicidal adolescents, incorporating Brite[TM], a smartphone app that promotes emotion regulation and provides access to a personalized safety plan during the transition from inpatient to outpatient care, plus in-person treatment as usual	PER, TLR, ORT, F2F, REM, COA, SAC, SKP	Daily use for 3 weeks [19 mood ratings, 10 times adding content, 8.50 times removing content, and 21 times accessing contacts]	INT, APP	Clinical [Treatment as usual inpatient care focused on diagnosis, safety assessment, stabilization, pharmacotherapy, psychoeducation, and disposition]

Study	Age Range *M(SD)*	*N*	Sample Characteristics	Intervention Description	Intervention Features	Prescribed [Completed][a] Dosage (Frequency, Duration) *Average unless otherwise noted*	Outcome Types	Comparison Group(s)
Kenny, 2016; Kenny et al., 2016; 2020 [159-161]	15-18 16.05 (0.76)	569	Irish predominantly White youth in 4th to 6th year of secondary school	CopeSmart, a smartphone app promoting self-management via positive coping strategies and increased emotional self-awareness (ESA)	PER, REM	Daily for 4 weeks [App used for 5 days during 28-day intervention period]	GWB, INT, SKL	No intervention
Kollei et al., 2017 [162]	18-31 21.65 (2.92)	53	German university students with elevated body dissatisfaction	Mindtastic Body Dissatisfaction (MT-BD), a cognitive-behavioral smartphone app that trains participants to avoid dysfunctional stimuli and approach functional stimuli	PER, GAM, ORT, F2F	5 minutes daily for 2 weeks, plus 3 face-to-face sessions [Not reported]	INT, OMH	No intervention [Waitlist]
Lee et al., 2013 [163]	1-10 5.07 (2.01)	80	Korean children undergoing general anesthesia for an elective surgery	Seven game-based smartphone app options serving as behavioral interventions for reducing preoperative anxiety	--	1 session for 5-10 minutes [Assume completed as prescribed]	INT	Clinical [Received an intravenous injection of 0.15 mg/kg midazolam]
Lee et al., 2018 [164]	16-47 20.90 (--)	206	Canadian university students	DeStressify, a mindfulness app that delivers mindfulness-based exercises including grounding visualization, gratitude, and finding meaning	REM	5 days a week for 3-23 minutes for 4 weeks [Self-report only: When adherence was self-reported using a scale from 0-10, the mean was 6.36]	GWB, INT, HTH, OTH, APP	No intervention [Waitlist]
Levin et al., 2020 [165]	18-25 20.44 (2.47)	23	United States predominantly non-Hispanic White, female university students seeking treatment at the counseling/psychological services center	Stop, Breathe, and Think, a mindfulness meditation-based smartphone app	TLR, GAM	Use daily for 4 weeks [Self-report only: Among 80% of participants who self-reported usage data at mid-intervention, usage occurred on 7.44 days over the past 2 weeks. Among 50% of participants who reported usage data at post-intervention, usage occurred on 5.80 days over the past 2 weeks]	GWB, INT, SKL, HTH, OTH, APP	No intervention [Waitlist]

Study	Age Range *M(SD)*	*N*	Sample Characteristics	Intervention Description	Intervention Features	Prescribed [Completed][a] Dosage (Frequency, Duration) *Average unless otherwise noted*	Outcome Types	Comparison Group(s)
Liguori et al., 2016 [166]	6-11 8.70 (2.35)	40	Italian youth undergoing a surgical intervention (eg, phimosis, abdominal hernia, orthopedic corrections)	Clickamico (Buddyclick), a tablet-device-delivered video providing psychoeducation about the surgical procedure to reduce preoperative anxiety	--	1 session for 6 minutes [Assume completed as prescribed]	INT	Inert [Answering parent/child questions about surgical procedure]
Marx, 2016 [167]	18-30 22.11 (3.29)	96	United States female undergraduate and graduate students with eating or weight concerns	Mindful Eating Coach, an app with roots in appetite awareness training (AAT) and appetite monitoring (AM), which aims to train individuals to coach themselves to eat more mindfully	PER, ORT, REM	Use as much as feasible for 3 weeks [2.44 appetite ratings per day over the modal number of days rated, 22 days]	SKL, HTH, APP	No intervention [Waitlist]
Mason et al., 2016 [168]	14-18 16.20 (1.39)	30	United States diverse sample of adolescents with moderate dependence on nicotine	Mobile device text messaging intervention, designed within the framework of motivational interviewing	PER, TLR, ORT, REM, COA, SAC	6 texts per day, for a total of 30 texts over the course of 5 days [Those who finished the trial completed 81.50% of all responses to the texting intervention]	SKL, HTH, OTH, APP	Inert [Health-based texts]
Mason et al., 2018 [169]	18-25 20.80 (2.47)	30	American young adults meeting DSM-5 criteria for cannabis use disorder	PNC-txt (Peer Network Counseling) text messages incorporating motivational interviewing and a relational framework	TLR, ORT, REM, SAC	112 texts, 20 minutes total, over the course of 4 weeks [Completed 99.40% of responses]	HTH, OTH	No intervention
McCloud et al., 2020 [170]	18-54 24.30 (6.71)	168	United Kingdom university students with elevated depression and/or anxiety symptoms	Feel Stress Free, a self-guided smartphone app incorporating cognitive-behavioral techniques and mindfulness-style meditation	--	At least 10-minutes on 1 or more activities, once a week for 6 weeks [Self-report only: Of participants who completed all follow-up questionnaires and self-reported outcome data, 80% indicated using the app weekly or more frequently]	INT, APP	No intervention [Waitlist]

Study	Age Range *M(SD)*	*N*	Sample Characteristics	Intervention Description	Intervention Features	Prescribed [Completed][a] Dosage (Frequency, Duration) *Average unless otherwise noted*	Outcome Types	Comparison Group(s)
Moore et al., 2013 (Study II) [171]	--- 22 (3.66)	43	United Kingdom university students	Intervention and comparison participants received daily texts for 2 months asking about alcohol consumption. Only intervention participants received a text midway estimating how much they spent on alcohol, intended to reduce their use	TLR	Participants instructed to respond to daily text messages asking about alcohol consumption [98.85% of intervention participants responded at least once to the daily text messages; 40% of participants responded each day]	HTH	Inert [Daily SMS or text messages requesting alcohol consumption data from preceding day (same procedure as intervention group)]
Newman et al., 2021 [172]	18-42 21.40 (---)	100	United States undergraduate students with self-reported excessive worry, anxiety, or stress	Lantern, a smartphone-based app delivering primarily cognitive and behavioral techniques to reduce generalized anxiety disorder (GAD) symptoms	REM, COA, SAC, SKP	40, 10-minute sessions divided into 8 units [12.38 sessions, 4.89 minutes each session over 11.52 weeks]	GWB, INT, APP	No intervention
Nguyen-Feng, 2019 [173]	--- 21.30 (4.20)	382	United States undergraduate students experiencing stress and wanting to learn how to better manage their stress	Ecological momentary intervention (EMI), a transtheoretical app incorporating written exercises, mindfulness exercises, and videos intended to reduce psychological distress	ORT, REM	Twice daily over the course of 14 days [22.50 surveys completed and 12.83 pages viewed over the course of 2 weeks]	GWB, INT, SKL, APP	Inert [Ecological momentary assessment brief online surveys with self-monitoring]
Nolan, 2019 [174]	18-31 21 (---)	89	United States premedical and nursing students at a southern state university	Headspace mindfulness-based app (specifically, the Take Ten program, which involves ten daily ten-minute guided meditation sessions)	ORT, REM	10, 10-15-minute sessions, conducted daily for approximately 10-15 days [Of the 33 participants assigned to the treatment group who completed pre- and post-measures and at least 1 session, 10 completed all 10 sessions, 24 completed 5 or more sessions, and 9 completed 4 or fewer sessions]	GWB, INT, SKL, APP	No intervention

Study	Age Range *M(SD)*	*N*	Sample Characteristics	Intervention Description	Intervention Features	Prescribed [Completed][a] Dosage (Frequency, Duration) *Average unless otherwise noted*	Outcome Types	Comparison Group(s)
O'Dea et al., 2020 [175]	12-16 14.82 (0.94)	193	Australian adolescents	WeClick, a smartphone app incorporating a framework of both cognitive-behavioral theory and social learning theory	PER, TLR, REM	Single-session intervention taking 1 hour to complete [68.10% completed all components of the intervention, with a completion time of 5:55 minutes, and a total of 19 minutes on the app]	GWB, INT, SKL, OTH, APP	No intervention [Waitlist]
Orosa-Duarte et al., 2021 [176]	--- 23.00 (4.16)	154	Spanish college and graduate students in the medical field (ie, nursing, medical school, psychology)	REM (Regulación Emocional basada en Mindfulness) Volver a Casa, a mindfulness-based smartphone app designed to reduce anxiety and increase empathy	--	8 stages, with a total of over 200 minutes of sessions, to be completed 1 stage per week over 8 weeks [Not reported]	INT, SKL	**Group 1:** No intervention **Group 2:** Clinical [In-person mindfulness-based program]
Parisod et al., 2018 [177]	10-13 11 (---)	151	Southwest Finnish early adolescents	Fume, a health education app including information on the topic of tobacco	PER, GAM, ORT	Use as much as feasible for 2 weeks [4, 19-minute sessions for 3 days]	HTH, OTH, APP	**Group 1:** No intervention **Group 2:** Inert [Non-gamified website intervention]
Patel et al., 2006 [178]	4-12 6.84 (0.40)	112	American children undergoing general anesthesia for an elective surgery	A hand-held video game using cognitive and attentional distraction techniques	GAM	1 session conducted at least 20 minutes prior to surgery [Assume completed as prescribed]	GWB, INT	**Group 1:** Clinical [Parent presence and oral midazolam] **Group 2:** Clinical [parent presence]
Pbert et al., 2020 [179]	--- 16.90 (1.10)	146	American adolescents in grades 9-12 with moderate dependence on nicotine (ie, smoked at least 5 cigarettes a day for the past 7 days)	Craving to Quit (C2Q), a mindfulness-based smartphone app designed to reduce smoking	TLR, REM, COA, SAC	22 modules, 5-15 minutes to complete each, over the course of 4 weeks [61% of modules were completed, 77% of participants used the app]	HTH, APP	**Group 1:** Clinical [NCI app, designed to reduce smoking] **Group 2:** Inert [Written smoking cessation materials]

Study	Age Range *M(SD)*	*N*	Sample Characteristics	Intervention Description	Intervention Features	Prescribed [Completed][a] Dosage (Frequency, Duration) *Average unless otherwise noted*	Outcome Types	Comparison Group(s)
Pierce, 2019 [180]	18-58 21.76 (5.92)	106	United States undergraduate and graduate students with symptoms of anxiety and depression who were interested in improving their mental health	An app based on the ACT (Acceptance and Commitment Therapy) Matrix, focused on building tacting of function (TOF) as an intervention for psychological inflexibility	ORT, REM	3, 15-20-minute online sessions, conducted once per week over 3 weeks, in addition to daily app use, followed by app use as-needed for the following 5 weeks [79.17% of participants completed all 3 online intervention sessions and 74.07% used the app at least once]	GWB, INT, SKL, APP	No intervention [Waitlist]
Piskorz & Czub, 2018 [181]	7-17 11.42 (3.42)	38	Polish children hospitalized for kidney dysfunction	A VR game designed within the principles of the mobile object tracking (MOT) task, to be used with a head-mounted Oculus DK2 HMD VR device	GAM, ORT	1 session, for approximately 5 minutes [Assume completed as prescribed]	GWB, HTH	Clinical [Treatment as usual]
Piskorz et al., 2020 [182]	7-17 12.42 (2.52)	57	Polish children hospitalized for kidney dysfunction	A VR game designed within the principles of the mobile object tracking (MOT) task, to be used with a head-mounted Oculus DK2 HMD VR device	GAM, ORT	1 session, for approximately 10-15 minutes [Assume completed as prescribed]	GWB, HTH, APP	Clinical [Treatment as usual]
Ponzo et al., 2020 [183]	18-25 19.61 (1.79)	262	United Kingdom university students with elevated levels of stress and anxiety	BioBase smartphone app and BioBeam wrist-worn wearable device incorporating elements of CBT, biofeedback, behavioral activation, and mindfulness	PER, ORT	At least 5 minutes a day, over 29 days [21.90 days, 7.06 minutes per day of use, or 5.33 minutes per day of trial]	GWB, INT, APP	No intervention [Waitlist]
Ranney et al., 2018 [184]	13-17 14.94 (1.18)	116	United States diverse sample of adolescents presenting to the emergency department (ED) and reporting past-year physical peer violence and depressive symptoms	iDOVE, a brief computerized intervention followed by a text messaging curriculum, used on a mobile device, combining the elements of motivational interviewing and CBT	TLR, ORT, F2F	1, 15-20-minute brief computer-guided intervention followed by 8 weeks of daily text messages [22 minutes for the computer-guided portion. For the mobile intervention, 96% responded to at least 1 of the daily text messages, with 47 responses per participant]	INT, APP	Inert [Healthy lifestyle control group, received an information-based, computer-guided intervention followed by health-based text message content twice a week]

Study	Age Range *M(SD)*	*N*	Sample Characteristics	Intervention Description	Intervention Features	Prescribed [Completed][a] Dosage (Frequency, Duration) *Average unless otherwise noted*	Outcome Types	Comparison Group(s)
Rodgers et al., 2005 [185]	16 and above 25 (---)	1,705	New Zealand ethnically diverse residents over the age of 15 who currently smoked cigarettes	Mobile phone text messaging intervention incorporating the principles of CBT	PER, TLR, SOC, COA, SAC, SKP	For 6 weeks participants received 5 texts a day. After 6 weeks, participants received 3 texts per week until the end of the 26 weeks [Not reported]	HTH	No intervention [Waitlist]
Rodgers et al., 2018 [186]	14-19 18.36 (1.34)	274	United States predominantly female adolescents and emerging adults, including high school and first-year college students	BodiMojo, a mobile app incorporating mindfulness, self-kindness, and common humanity, intended to promote positive body image through self-compassion	ORT	Twice daily for 6 weeks [Not reported]	GWB, SKL	No intervention
Sandrick et al., 2017 [187]	18-30 19.40 (1.00)	60	American college students	A mobile-phone text messaging intervention designed with motivational and action-oriented texts	TLR, F2F, COA, SAC	Text messages were scheduled for Tuesdays, Thursdays, and Saturdays for 8 weeks [87.31% (2187/2505) of messages were viewed by intervention participants]	GWB, HTH	Inert [Behavioral assessment at intake, but lacked coaching portion or text messages]
Siembor, 2017 [188]	18-25 20.89 (2.03)	24	United States racially diverse sample of college and graduate students	The Mindfulness App, a mindfulness-based smartphone app	TLR, REM	Required to complete at least 15 minutes a day, totaling 90 minutes a week for 4 weeks [Not reported]	GWB, SKL, APP, OTH	No intervention
Suffoletto et al., 2014; 2015 [189,190]	18-25 21.93 (2.03)	569	United States racially diverse sample of emerging adults with previous hazardous alcohol use, but not currently seeking alcohol treatment	Text messaging intervention incorporating motivational enhancement and tailored feedback intended to reduce binge drinking	TLR	Twice per week for 12 weeks [81% of participants responded to text queries on Thursdays and 77% responded on Sundays]	HTH	No intervention

Study	Age Range *M(SD)*	*N*	Sample Characteristics	Intervention Description	Intervention Features	Prescribed [Completed][a] Dosage (Frequency, Duration) *Average unless otherwise noted*	Outcome Types	Comparison Group(s)
Suffoletto et al., 2012 [191]	18-24 21.00 (1.80)	45	United States racially diverse young adults identified as hazardous drinkers	Text messaging intervention with goal setting, intending to reduce heavy drinking by collecting drinking data and providing immediate feedback and ongoing support	TLR, ORT, REM	Once per week text message queries for 12 weeks [80% of participants in the intervention group completed all 12 weeks of queries]	HTH	**Group 1:** No intervention **Group 2:** Inert [Text message drinking assessment group (without feedback)]
Tennant et al., 2020 [192]	7-19 11.59 (3.34)	93	Australian child and adolescent oncology patients	Immersive virtual reality, to be used on a VR headset (Samsung Gear VR®) connected to a smartphone	ORT	1, 10-minute virtual simulation experience [61 participants completed the single-session intervention]	GWB, INT, OMH, HTH, APP	Clinical [iPad condition presented identical content, without the immersive experience]
Tighe et al., 2017 [193]	18-56 26.25 (8.13)	61	Australian, primarily Aboriginal "youth," with elevated depression or psychological distress, some in conjunction with suicidal ideation	Ibobbly, an acceptance-based therapy app targeting suicidal ideation that contains three content modules and three self-assessments	PER, ORT, F2F, COA	6 sessions (3 content modules and 3 self-assessments) to be conducted about once per week for 6 weeks [Among the 66% of participants who uploaded their usage data, 85% completed all 6 sessions]	GWB, INT, SKL, APP	No intervention [Waitlist]
Vu, 2018 [194]	18 and above ---	420	American college students	Pacifica, an app that incorporates cognitive-behavioral and mindfulness techniques, intended to address anxiety and low mood	TLR, REM	Daily for 2 weeks [61% used "Relax" activities at least once (2.10 times on average). 48% used "Thought Entry" and 18% used "Goals Entry"]	GWB, INT, SKL, APP	**Group 1:** No intervention [Waitlist] **Group 2:** Inert [Pacifica Lite app, attention placebo without psychotherapy-based components]
Wang et al., 2020 [195]	--- 16 (---)	114	Taiwanese vocational students	**Intervention group 1:** Sweet Dreams in Taiwan, a social-cognitive theory-based mobile community game aimed at transforming cognitive change into behavioral change through the practice of healthy sleeping habits, with a traditional, in-person sleep hygiene course	PER, TLR, SOC, GAM, ORT, F2F, REM, COA, SAC, SKP	Daily use over 12 weeks [Not reported]	HTH, OTH	Inert [In-person, traditional sleep hygiene course and sleep sensor feedback]

Study	Age Range *M(SD)*	*N*	Sample Characteristics	Intervention Description	Intervention Features	Prescribed [Completed][a] Dosage (Frequency, Duration) *Average unless otherwise noted*	Outcome Types	Comparison Group(s)
				**Intervention group 2:** Sweet Dreams in Taiwan (same as above), in addition to an in-person, social-cognitive-based sleep hygiene course				
Wantanakorn et al., 2018 [196]	5-12 9.00 (3.25)	60	Thai youth undergoing bone marrow aspiration procedures	Children-Friendly Hospital, an app providing information about the bone marrow aspiration procedure and a game to help children cope with anxiety	GAM	Single-session intervention [Assume completed as prescribed]	INT	Inert [Verbal information about procedure protocols]
Whitehouse et al., 2017 [197]	--- 3.38 (0.69)	80	Australian children with an autism spectrum disorder diagnosis	Therapy Outcomes By You (TOBY) iPad app, using Applied Behavior Analysis (ABA) principles to teach visual and auditory understanding, imitation, receptive and expressive language, and social skills	TLR, ORT, REM, COA, SAC, SKP	Daily use for 20 minutes per day over 6 months [19 minutes per day during the first 3 months, but only 2 minutes per day during the second 3 months]	OMH	No intervention
Whittaker et al., 2012; 2017 [198,199]	13-17 14.30 (0.90)	855	New Zealand (14.30% Māori or Pasifika) students in grades 9-12 without elevated or diagnosed depression	MEMO, a mobile website intervention using cognitive-behavioral therapy tools to build positivity and prevent depression	ORT	2 messages daily for 9 weeks [19% saw at least half the messages]	GWB, INT, APP	Inert [Mobile phone messages on healthy eating, information about cyber safety and environmental tips]
Yap et al., 2020 [200]	18-21 18.66 (0.70)	176	Filipino college students who drank alcohol at least once in their lives	Drug Defense, a cognitive-behavioral app intended to increase adolescents' knowledge on alcohol use and decrease intent to use and actual alcohol use	GAM	1, 20-40-minute single-session intervention [Assume completed as prescribed]	HTH, OTH	Inert [Video documentary]

Notes. *N* = Number of participants (within included groups) randomized at pre-intervention. eBAC = Estimated blood alcohol concentration. CBT = Cognitive-behavioral therapy. VR = Virtual reality. IEP = Individualized education program. **Intervention technological and support features**: PER = Personalization, TLR = Tailoring, SOC = Social component, GAM = Gamification, ORT = Orientation, F2F = Other in-person (face-to-face) element, REM = Reminders, COA = Human or bot support or coaching, SAC = Supportive accountability, SKP = Supervised skills practice. **Outcome types**: GWB = General psychological well-being or distress, INT = Internalizing (depression and/or anxiety), OMH = Other (non-internalizing) mental health problem, SKL = Psychosocial strategies or skills, HTH = Health [behavior], OTH = Other (eg, academics, interpersonal relationships, parenting strategies or symptoms, psychology of health-related knowledge), APP = App (mTDI) or intervention ratings, or data pulled from app. Further details in Methods.

[a]Self-reported usage is noted only in cases of studies reporting self-reported but not objective user statistics.
